# Factors Affecting Patient and Physician Engagement in Remote Health Care for Heart Failure: Systematic Review

**DOI:** 10.2196/33366

**Published:** 2022-04-06

**Authors:** Ahmed Al-Naher, Jennifer Downing, Kathryn A Scott, Munir Pirmohamed

**Affiliations:** 1 Institute of Systems Molecular and Integrative Biology University of Liverpool Liverpool United Kingdom

**Keywords:** remote care technology, chronic adult heart failure, qualitative synthesis, thematic analysis, patient compliance, patient engagement, elderly population, carers, health care professionals, technology implementation

## Abstract

**Background:**

Adult chronic heart failure mainly affects an elderly population with multiple comorbidities that often require frequent medical visits to prevent poor health outcomes. However, the heart failure disease process reduces their independence by reducing mobility, exercise tolerance, and cognitive decline. Remote care technologies can bridge the gap in care for these patients by allowing them to be followed up within the comfort of their home and encourage their self-care. However, patients, carers, and health care professionals need to engage with the technology for it to be useful.

**Objective:**

This systematic review explores qualitative primary studies of remote care technologies used in heart failure, to determine the factors that affect user engagement with the technology. This is explored from the perspective of patients, carers, and health care professionals.

**Methods:**

Relevant studies published between January 1, 1990, and September 19, 2020, were identified from EMBASE, Ovid MEDLINE, PubMed, Cochrane Library, and Scopus. These studies were then synthesized using thematic analysis. Relevant user experiences with remote care were extracted using line-by-line coding. These codes were summarized into secondary codes and core concepts, which were further merged into overarching themes that encapsulate user experience with remote care.

**Results:**

The review included 47 studies, which led to the generation of 5 overarching themes that affect engagement: (1) “Convenience” relates to time saved by the intervention; (2) “Clinical Care” relates to perceived quality of care and health outcomes; (3) “Communication” involves feedback and interaction between patients, staff, and carers; (4) “Education” concerns the tailored information provided; and (5) “Ease of Use” relates to accessibility and technical barriers to engagement. Each theme was applied to each user base of patient, carer, and health care professional in a different manner.

**Conclusions:**

The 5 themes identified highlight aspects of remote care that facilitate engagement, and should be considered in both future design and trials evaluating these technologies.

## Introduction

The majority of people living with heart failure are elderly and have 3-5 severe comorbidities such as diabetes mellitus, chronic obstructive pulmonary disease, and ischemic heart disease [1]. The 1-year mortality from hospital admission is more than 30%, and 30%-50% of people living with heart failure are re-admitted to hospital each year [2]. Heart failure often leads to reduced mobility and shortness of breath on exertion. These manifestations can affect day-to-day living, reduce independence, increase the risk of falls, and impair access to health services [3].

Remote care technologies have the potential to benefit people living with heart failure by bridging the gap between clinical visits [4]. These technologies enable frequent observations in the home and give access to tools to aid self-care [5]. In the long term, personalized, community-based technology may reduce the burden of health care appointments and increase effective management of symptoms. Empowering self-management can enable early detection of health issues and promote interventions to prevent hospital admission [6]. However, only technologies which engage the users (including carers) can produce positive change. Engagement depends on the design and suitability of the technology to the cohort. Similarly, effective interventions must also engage health care professionals [7].

Non-engagement with health care technologies is widespread [7]. A meta-ethnography by Greenhalgh and colleagues [8] investigating technology-supported health programs generated the Non-adoption, Abandonment, Scale-up, Spread, Sustainability (NASSS) framework, which identifies 7 critical domains that impact whether a technology is adopted. These are the condition, the technology, the value proposition, the staff involved, the organization, the social/institutional context, and the interaction between domains. This approach gives valuable insights into the dynamic between users and medical technologies in general, but a more nuanced approach is needed to understand interventions in patients with heart failure having complex needs.

For instance, in the review by Simblett et al [7] regarding barriers to engagement of remote technology, a large proportion of dropouts from remote care studies were attributed to usability issues such as technical difficulties, over 5 times more than those that dropped out due to issues with their health status. In an unselected population this may be representative; however, in the cohort of patients with heart failure, patient barriers and priorities for engagement can be very different. Elderly populations are likely to have impairments in vision, dexterity, and hearing, which can impact usability in different ways. In addition, the higher incidence of comorbidities may mean more hospital admissions and variability in their health status. Therefore, a focused approach is needed to determine which factors play a greater role for engagement with this particular patient group so that future interventions can target these areas and incorporate the design elements that matter most. Otherwise, there is a risk of creating an intervention that is not well suited to the target population, which can lead to these large numbers of dropouts and disengagements.

The aim of this systematic review was to explore the use of remote distance technologies in heart failure. We focused on the effect that remote distance technologies have on engagement with care and investigated the perspectives of people living with heart failure, carers, and health care professionals interacting with the same technology. Our aim was to identify the unique contexts and issues found in this cohort, focusing on factors that influence adoption of, engagement with, and use of remote care interventions by people living with heart failure; and factors that affect the engagement of clinical staff with remote care intervention. We also considered which of the tensions identified in clinical trials became evident from examining patient and staff personal experiences.

## Methods

### Study Selection

Relevant studies published between January 1, 1990, and September 19, 2020, were identified from EMBASE, Ovid MEDLINE, PubMed, Cochrane Library, and Scopus (see Multimedia Appendix 1 for search history). Reference lists of all identified studies were manually searched for relevant publications.

### Inclusion Criteria

- Primary studies using qualitative methodologies to collect or analyze data—defined as studies where participants can enter free-text comments or answer at least one open interview question.
- Studies that included patients diagnosed with adult chronic heart failure of all severities or their carers or the health care professionals involved in their care.
- Studies describing remote care programs—defined as any intervention accessible from the patient’s home or local community, which provides the patient with education, assessment, investigation results, or otherwise replaces a service that would normally be offered within a formal clinical setting.

### Exclusion Criteria

- Nonprimary studies.
- Studies with no qualitative element in the collection or analysis of data.
- Studies that did not include patients with adult chronic heart failure and neither their carers nor health care professionals.
- Non-English studies
- Interventions already established for heart failure care within national/international guidelines.
- Interventions involving implantable devices where user engagement is not a factor.

A random sample of 30% of abstracts were screened independently by 2 reviewers (AA and JD). The remaining abstracts were selected by a single reviewer (AA) using the double-screened sample as a foundation. Full texts were assessed for eligibility by 2 reviewers (AA and JD). Study characteristics were extracted using the Enhancing Transparency in Reporting the Synthesis of Qualitative Research (ENTREQ) statement [9], and with guidance from the Consolidated Criteria for Reporting Qualitative Research (COREQ) checklist [10]. We used the PROGRESS-Plus framework (ie, *p*lace of residence, *r*ace, *o*ccupation, *g*ender, *r*eligion, *e*ducation, *s*ocioeconomic status, *s*ocial capital, plus personal characteristics/relationships/time-dependent variables) [11] to create a more comprehensive review guideline for identifying health inequalities and characterizing populations [12]. Quality assessment of these studies ensured that the findings extracted were reliable and that bias was minimized [13]. Methodology of included literature was assessed using The National Institute for Health and Care Excellence (NICE) Quality Appraisal Checklist for Qualitative Studies [14-16]. Studies were designated excellent (++), good (+), or poor (–) quality. Rather than excluding the poor-quality studies, we opted for a more inclusive study design to better encompass the range of user experiences and reach a saturation point for first-order codes.

### Thematic Synthesis Methodology

We applied the thematic analysis model of qualitative evidence synthesis [17]. Textual data from the *Results* or *Findings* sections of publications were extracted verbatim (EPPI-Reviewer 4, version 4.7.1.2) and line-by-line coding was used to generate first-order codes. First-order codes were grouped under second-order codes—umbrella terms that bridged commonalities between multiple first-order codes. Second-order codes were created independently by AA, JD, and a public advisor group, and the final list was determined by iterative consensus. Third-order, “core concept” codes were created based on patterns and inferences observed throughout the review. They involved a process of reflection and reiteration by the authors on all previously extracted data and codes within the context of the research question being asked, that is, what are the factors affecting engagement.

Core concepts and second-order codes were finally consolidated to create overarching themes, which were designed to be universal across patients, health care professionals, and carers, and to encapsulate all aspects of experiential user engagement. A patient participation group made up of several patients with heart failure was vital in establishing meaningful nomenclature for each of the themes and what they encompass. The title of each theme would then be intended to be interpreted in the context of its underlying description, based on its included secondary codes and core concepts, making it a unique thematic construction. At all stages of the review, the authors reflected on their own background and position and how it would affect the design, analysis, and interpretation of the research conducted [17].

## Results

### Study Selection

**Figure 1:**
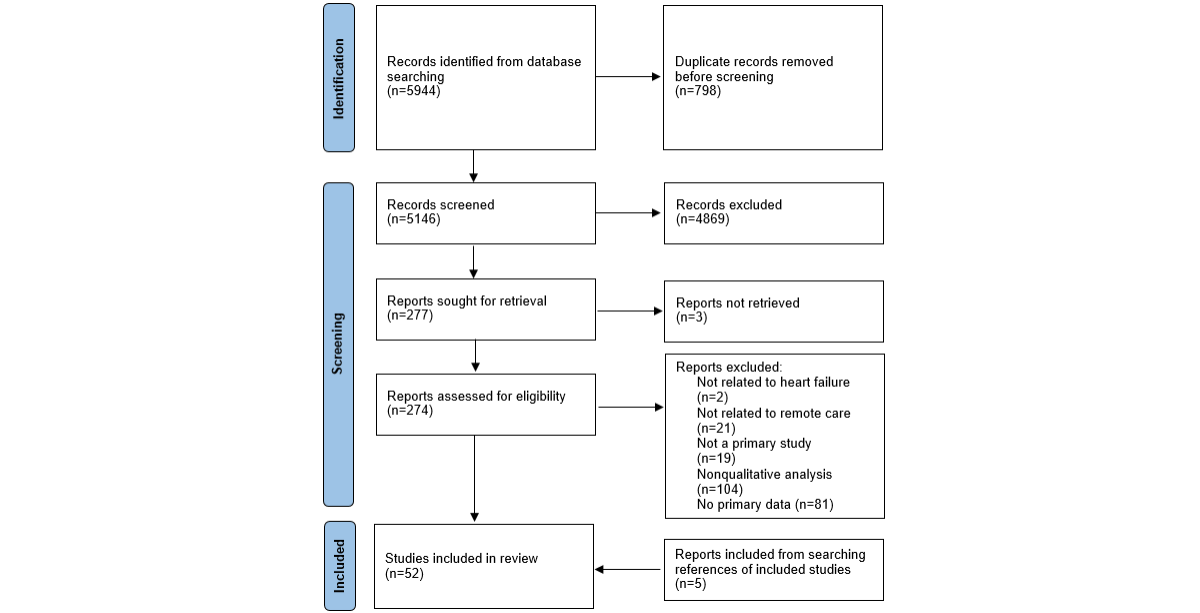
PRISMA flow diagram for included studies. PRISMA: Preferred Reporting Items for Systematic Reviews and Meta-Analyses.

Our initial search criteria found 5944 matching studies, of which 798 were duplicates and 4869 did not match the inclusion criteria based on their title and abstract. A further 230 studies failed to meet the inclusion criteria when full texts were screened. The remaining 47 studies were included in the full review, in addition to 5 studies referenced by these papers (Figure 1). The PRISMA checklist (Multimedia Appendix 2) was used to analyze the included studies. Fifty-two studies were thus included in the final review (see Multimedia Appendix 3 for characteristics of studies table). The rationale for quality assessment scores is described in Multimedia Appendix 4.

A total of 33 studies covered remote monitoring systems used to review a patient’s status from their home [18-50]. Clinical decision tools, used to complement clinician management plans, were trialed in 4 studies [23,51-53]. As many as 4 studies involved patient health information platforms [23,54-56], 5 online patient self-management tools via an online portal, [23,56-59], and 4 educational tools delivering information for self-care through various means [23,58,60,61]. Community remote care, involving occasional home visits by nurses, comprised 2 studies [62,63], as did telephone consultations [48,64]. There was 1 instance each of a peer-support system [65] and a pharmacy-based consultation [66]; 2 studies delivered a concept of a remote care intervention and gathered opinions based on a theoretical design [46,67].

People living with heart failure, carers, and health care professionals are not equally represented in these sources: 9 studies included perspectives from health care professionals alone [31,37,39,44,45,48,51-53]; 29 included patients alone [18,19,21-24,28-30,33-35,38,40,41,46,47,54,56,57,59,60,62-65,67-69]; 2 included patients and their carers [20,58]; 9 included patients and health care professionals [26,32,36,42,43,49,55,61,66]; and 3 included health care professionals, patients, and carers [25,27,50].

### Themes

#### Overview

**Figure 2:**
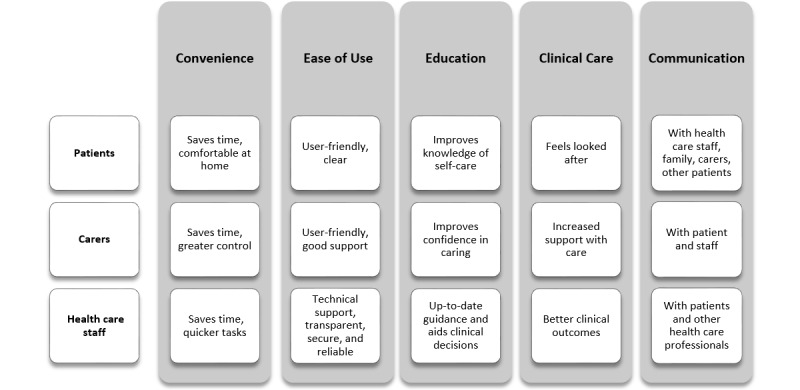
Final thematic map representing the key points of each engagement theme and what they mean to each user group.

We extracted 110 separate primary descriptive codes from the *Results* sections of the included studies (Multimedia Appendix 5). We used a combination of grouping and reflective review to categorize these themes, which resulted in 30 secondary codes (Multimedia Appendix 6) and 13 core concepts. Secondary codes and core concepts were synthesized to give 5 themes that seek to encapsulate all aspects of user engagement: Convenience, Ease of Use, Education, Clinical Care, and Communication (Figure 2). Although themes are applicable across patients, carers, and health care professionals, they were associated with different secondary codes and core concepts.

#### Convenience

Convenience is any aspect of the intervention that makes the user’s life easier than the preintervention stage, by either time-saving or giving them the freedom to do more, or enhances their comfort in the current environment.

Positive patient experiences included those where the intervention had saved the patient travel time, or opened up new options for the patient to manage their care at home, and be comfortable in their home environment [20]. Negative experiences were associated with extra work, for example, in a complex self-monitoring system that takes time away from daily activities [35]. Loss of control was also commonly reported as a negative experience [35]:

I think you feel like you're not in control of your life...I just felt that, well, it certainly wasn't for me, and to, from how he explained it, um, you tended to have to do your blood test every single day...I try to be a bit more relaxed and...I just felt it, it did put a bit more pressure on me...you know, holidays or if I had to stay at my mum's, oh God, I've got to come home and do the machine.

ID31

For carers, positive aspects related to increasing options for managing care and increased freedom. Most carer experiences fell into negative codes regarding losing control of their tasks and increasing their workload and hence their stress. Throughout this theme, the concepts of workload, responsibility, and stress correlated with each other with regard to trialing a new intervention.

Positive experiences for health care professionals related to time-saving through faster and more efficient decision making. An example from Taylor and colleagues [37] showed how remote care technologies can help in understaffed situations:

We are being asked to see more patients with no additional resources.... How can we release a little bit of our capacity? Because our capacity is at absolute maximum all the time...I think telehealth helps from that point of view

District Nurse 4, Site A

Negative experiences related to the workload associated with implementing the intervention. After using the intervention, some health care professionals also reported that patients had become too dependent on the staff via the increased monitoring they provided [25]. Many clinical professionals were also reluctant to change as new methods were seen to restrict their preferred working pattern [39].

#### Ease of Use

This refers to the technical design elements of the intervention, and how user-friendly and accessible it was.

Positive patient experiences were associated with an easy-to-use intervention, with simple instructions and very few technical difficulties. Patient negative experiences involved devices that were intrusive, difficult to use, and had many technical difficulties. High costs and unreliability of the intervention made some technologies “less accessible.” For example, Agrell and colleagues [18] described a remote care home hub with integrated stethoscope and blood pressure cuff, which proved difficult for sick patients to use.

Positive carer experiences centered around technology that was easy to use (eg, had a clear user interface) and reliable. Much of the negative feedback from carers was related to the stress caused by technical malfunctions of the device [54].

Health care professionals were positive about interventions that were easy to administrate and explain to patients and staff, or provided information in an intuitive manner. They favored interventions that took less time and effort to install and establish into current practice, such as a system that integrates remote monitoring into existing electronic patient records [52]. Negative feedback from health care professionals was associated with high cost, lack of security, poor user interface, lack of training provision, and unreliable results. An added issue for health care professionals was the lack of support and technical expertise to deal with problems [44].

#### Education

This relates to the ability of the intervention to provide appropriate, user-tailored information.

Positive patient experiences were reported in interventions that delivered self-care information in a simple, structured format, which was easy for patients to make use of. This helped increase their confidence in managing their condition, which in turn increased their engagement [55,64]. Negative patient experiences occurred where either useless or irrelevant information was given. This included remote care devices that give no immediate feedback, and websites or electronic health records that used medical jargon [55].

For carers, it was important to learn about the condition of the patient and understand disease progression. It was also important to access up-to-date information on the status of the patient. Remote interventions that made this easy for them generated positive feedback. When this information was difficult to access or mired in inaccessible jargon, it led to disengagement and discontentment [54].

For health care professionals, education centered on informative benefit that an intervention added to current practice. Positive experience involved interventions that provided guidelines or clinical suggestions to aid in patient management. Clinicians also identified the educational benefit on self-care habits of patients [27]. Education provided through a remote care intervention raises the standard of clinical treatment and involves the patient in their own management. This continuous benefit is valued by health care professionals. By contrast, the negative experiences of education involved insufficient, irrelevant, or poor-quality clinical information [22]. An intervention that did not provide feedback or allow patients to be updated on their health status provides little motivation for using the device or improving self-care, and this had a knock-on effect on health care professionals’ perceptions of the usefulness of the device.

#### Clinical Care

This was a clearly defined theme that involved either improving or hindering the current clinical care provided for heart failure management. In most cases, the effects of this theme could be measured and observed in terms of patient outcome over the long term.

Patients valued interventions that supported better symptom control and improved confidence in their treatment management plan. They often commented that certain remote care interventions made them feel more “looked after” once they were aware that their measurements were being monitored from afar [22]. Negative experiences involved difficulty integrating the intervention into their daily lifestyle. Some patients felt that the intervention had no effect and that the technology was ultimately not needed [28].

Positive aspects for carers focused on them feeling more supported while using the intervention. The reassurance of extra clinical support helped them perform their task so that they felt they need not struggle alone [20,54]. Negative aspects of clinical care from carers were often found where patients already had sufficient care to meet their needs. Additionally, there were cases where the patients were too sick to be under an automated monitoring system and required personal supervision. Where carers mistrusted technology, its introduction was an extra source of stress [35].

For health care professionals, positive aspects involved being able to administer guideline-recommended management reliably and with less error. The ability to provide proactive treatment was also considered to be of great benefit. Often, increased monitoring frequency allowed the staff to identify deterioration more quickly and intervene earlier to prevent worsening outcomes. This improved the safety and quality of management and also fulfilled a “safety-netting” criteria that were found to be very valuable [22]. Remote care bridges the gap between primary and community care, where patients with heart failure are seen infrequently and may have periods of long stability, but may also be at risk of sudden deterioration. Negative aspects of clinical care for staff were equally impactful. They involved interventions that disrupted management, often by giving unreliable, incomplete, or false information, or simply made no difference to management decisions, or patient outcome. In the example by Sharma and colleagues [44], the remote care offered features such as home blood pressure monitoring, but did not provide enough information to assess for an infection. Health care providers perceived that some technology may hinder usual care in this way, and disrupt the current “face-to-face” care.

#### Communication

This theme encompasses the quality and frequency of interpersonal contact involved with the intervention.

When discussing communication, patients often referred to the frequency of contact with their nurse or doctor. In general, patients favored human contact to being monitored by a noninteractive device. Therefore, interventions that facilitated human contact appealed to patients [18,29]. Patients commented that greater human contact helped with their feelings of isolation and encouraged them to self-care. Remote care interventions that facilitated interaction with health care professionals were seen as valuable and worth the investment. Patients also liked interventions that could connect with their family and friends for further support. Some social interventions connected patients with each other and these showed widespread approval within this cohort of patients with heart failure. A chronic illness that reduces mobility often impacts the social activities of the patient and it is not surprising that this isolation can often be overlooked when considering these interventions [65].

Conversely, many of the remote care interventions led to reduced contact with physicians. Patients often missed the human contact element of regular clinic visits. Rather than having a device to provide information for them, they preferred being able to ask questions from staff members directly [18]. Without a human contact element, some felt more distant and perceived that something was lacking from the consultation. While remote care may obtain information efficiently, sometimes human contact is more reassuring and has a bigger impact on patient engagement.

For carers, “isolation” was predominantly related to the fear of leaving the patient on their own. The ability of remote care to mediate communication between both the patient and carer with health care professionals was seen as valuable and reassuring [26].

Health care professionals are often concerned with patient contact and with connection to specialists. Positive communication with other staff members is found in interventions that seamlessly connect multidisciplinary teams [37]. This in turn encouraged teamwork. Regarding staff to patient communication, health care professionals felt that frequent contact led to better awareness of the patient’s tendencies and hence earlier and better decisions on their care [27,31]. The increased availability of communication with patients via remote care was felt to increase trust between the staff and their patients and staff felt their patients were more open to them. This fosters a good clinical environment, especially in the management of chronic disease where familiarity with the patient is vital to detecting early deterioration. Increased communication opportunities also motivate both parties to continue to use the device, for reassurance and safety-netting. Negative staff experiences with communication included systems that did not connect with other team members or caused a disruption in teamwork. An example was a remote monitoring system that provided the general practitioner with extra information but was unable to send this information on to heart failure hospital consultants, and thus made referrals harder [22]. Staff also found that interventions that reduced interaction with patients received much less support from both parties. Reduced face-to-face time due to remote intervention created a sense of distance with the patient, which was concerning to some staff [22].

### Health Inequalities

#### Overview

In our review of interventions and the populations they were used on, various health inequality issues became apparent: usability, patient selection criteria, demographic distribution, and the potential to increase inequality gap through implementation of remote care.

#### Usability

In a heart failure cohort, interventions need to be usable by patients who have visual impairments or problems with dexterity [43]. If the intervention is not designed to take this into account, it can exclude the patients that might have benefitted the most. Riley and colleagues [34] recount an example of one such patient who struggled to use a remote care device, leading to undue stress:

I keep losing the finger contact and because of my sight I have to search hard to find it and that unfortunately sends my blood pressure up and then I have to redo the test.

Edward

In addition to co-ordination and visual problems, some patients were unable to use a weighing device because it required them to remain standing for several minutes [26].

#### Patient Selection

In any interventional trial, inequality may arise between patients who do and do not have access to remote interventions. Clinical decisions on which patients are most suited to the intervention can be subjective. The weight monitoring intervention in the study by Johnston and Weatherburn [26] demonstrated this selective service based on clinician opinion, creating a defined cohort of highly monitored patients above those of current care. With limited resources, clinicians may set up their own criteria for prioritizing access to remote care based on need, as they do with other treatments [22]. Over a short intervention trial period, some clinicians felt that monitoring was not useful in patients they deemed stable. However, this may lead to situations where patients are only provided with remote monitoring once deterioration has occurred.

#### Demographic Representation

In intervention trials, the criteria for proving the new remote care intervention are often decided a priori by the research group. Studies considered here show a distinct preponderance to White ethnicity and male gender. In addition, there is generally poor reporting on religion, socioeconomic status, social capital, disability, and vulnerable groups. It is therefore difficult to assess the impact of remote care integration on more disparate groups within populations.

#### Widening the Inequality Gap

Some clinicians showed anxiety that technology could widen the health inequality gap. The study by Earnest and colleagues [55] highlighted concerns around how remote care would translate in areas with fewer resources. Many remote care interventions take the form of highly technologically advanced, expensive devices, which, while rich with clinical data, are prohibitive for widespread use in terms of costs and resource drain. It is worth noting the impact of these interventions on the rest of the clinical care of the population, whether it creates a wider benefit to the whole or simply allows greater monitoring for those who have the most care in the first place.

## Discussion

### Principal Findings

Throughout the endeavor to define engagement with heart failure remote care by patients, carers, and staff, this review has encompassed a wide range of possible interventions and environments, exploring the qualitative experiences from a vast array of users until the point of saturation. The experiential discoveries have been organized into 5 overarching themes that can be applied to each user base: Convenience, Ease of Use, Education, Clinical Care, and Communication. These themes go some way to giving insight to designers of such technology on how to tailor their intervention and improve uptake to create a greater benefit for their intended users.

Recently, the advent of COVID-19 has led to the prioritization of remote care as a way of managing vulnerable populations such as these while reducing the risk of in-person exposure [70]. While technology usability models for remote care have been explored [7,8], there are yet many clinical studies specific to heart failure remote care that emphasize more research into engagement for this patient group [71,72]. Hence the themes generated in this review aid to bridge the gap between generic usability models and the heart failure population, helping to apply existing knowledge in a more personalized way for more engaging interventions [73].

### Implementing Engagement Themes Into Technology Design

Each theme has its own technological implications in the design of an intervention depending on the user group. Adding “Convenience” to a remote care intervention involves improving comfort or saving time in a way that has a significant impact on their daily life. This means that the user should be able to carry out typical tasks, such as patient self-care or a clinician’s daily reviews, but in a more efficient manner or at a location more convenient for both parties.

Adding “Ease of Use” involves tailoring the experience with the user in mind. For elderly patients with heart failure, this means a simple and intuitive interface, requiring little or no technical knowledge. The closer the intervention reflects normal daily activity, the more seamless the transition toward its use. For clinicians, interventions should provide the necessary information for clinical decision making without unnecessary complexity. Technical difficulties are some of the most important barriers to effective implementations of remote care. Technologies that are reliable, easy to install, and integrate into current systems will earn the trust of staff.

Improving the “Education” of an intervention involves allowing it to provide information in a relatable way that is specific to the user’s situation, for example, to aid patient self-care and to increase their sense of control and self-reliance [27]. For health care professionals, the intervention should help update staff on current guidelines and recommended practice. Information should be understandable and usable by all health care professionals from community nurses to heart failure specialists.

Adding the “Clinical Care” component for patients means improving the perception of greater care. These perceptions are enhanced by frequent feedback, contact with clinicians, and impactful changes to their lifestyle, which foster a sense of being “well looked after” [65]. For health care staff, clinical care relates to improving health outcomes. A robust series of clinical trials that demonstrate the clinical value of an intervention is vital to inform and justify implementation. Beyond this, clinicians will prioritize the interventions that address an unmet need in service provision. Overall, clinical care is a vital part of any remote care assessment, and must be assessed in the context of patients’ needs, their current care, and the resources available to the staff.

Improving “Communication” is achieved by allowing further communication to take place, both between patients and staff and between peers, fostering a sense of more involved care [25]. Additionally, many patients value the option of having remote care to connect to their support network. Health care professionals highly value mechanisms in remote care that allow multiple specialties and services to integrate together and communicate. Many people living with heart failure are elderly and have multiple comorbidities necessitating a complex regimen of medication and outpatient health services to maintain their well-being. Too often, the flow of patient information between these services becomes lost or confused. This is an obstacle to communication that well-designed remote care can help overcome.

### Implementation of Remote Care Within Clinical Trial Design

Based on our observations, we noted certain practices that need to be carefully considered during the design of remote care evaluation studies. Recorded experiences from users contrast and contradict each other even within the same study, and thus a sufficiently large sample size is important. The intervention must be distributed across a diverse ethnographical population, including those with poor literacy, and those with disabilities as well as diversity in gender and socioeconomic backgrounds. Selective inclusion or exclusion of patients with extremes of the condition may lead to bias. In our view, it is important to consider both the patient and health care professionals’ viewpoint in the same study. This gives a good estimate as to how readily staff were able to use the intervention, and the impact that it has on their current care. It is also important to know where in the established care pathway the new technological intervention will sit; this affects who administers the device, to which cohort, and through which electronic systems. Interventions that do not consider the gaps in current care may cause disruption for staff and create additional steps that do not complement nor aid their current care environment [35]. Finally, in a few cases, patients and staff had negative experiences with the third-party support provided around the device’s use. This could be improved with effective trial planning.

### Limitations

#### Limitations in Methodology

We have attempted to mitigate bias by double-coding, cross checking, and using grounded theory processes to construct a “blank-slate” perspective. However, in line-by-line coding some lines may have a double-meaning, fitting into multiple codes, and decisions on coding are subjective.

#### Limitations in Studies

The studies included were of a wide range of different methodological and analytical qualities. First-order codes from poor-quality studies may be subject to greater variability than high-quality studies. However, we included studies of all quality to avoid missing useful information and to achieve saturation. The wide range of study sizes may skew the experiences toward those that are mentioned in small sample sizes, as it increases the frequency of appearance of the related codes. We have listed the sample size of each study in the characteristics of studies table (Multimedia Appendix 1).

#### Limitations in Phenomena Studied

The researcher’s reflexivity plays a role in the overall generation of the themes and can lead to first-degree bias. This process happens both inductively and intuitively and it cannot truly be distanced from preconceptions and beliefs of the reviewers themselves.

### Conclusions

Overall, the 5 themes generated by this study overlap in many ways to create an engaging technology. Successful remote care interventions interact meaningfully with the user and thus instigate a change in self-care or in the working practice of health care professionals. Increased communication due to a remote care intervention leads to a perception of greater care from the patient. This in turn leads to improved feedback to the clinician and an improved perception of the devices’ educational and clinical benefits. Likewise, convenience can be an important component contributing to ease of use. Successful and engaging interventions should combine these 5 elements into their design to increase the engagement of their users and lead to a greater benefit in this elderly comorbid population that needs this support the most.

## Appendix

Multimedia Appendix 1 Search history for systematic review.

Multimedia Appendix 2 PRISMA 2020 checklist.

Multimedia Appendix 3 Characteristics of studies included with the first order codes generated from each study.

Multimedia Appendix 4 NICE quality appraisal.

Multimedia Appendix 5 First-order codes identified in studies included in systematic review, with associated descriptions.

Multimedia Appendix 6 Synthesis of second-order codes.

## Abbreviations

COREQ: Consolidated Criteria for Reporting Qualitative Research
ENTREQ statement: Enhancing Transparency in Reporting the Synthesis of Qualitative Research statement
NASSS framework: Non-adoption, Abandonment, Scale-up, Spread, Sustainability framework
NICE: The National Institute for Health and Care Excellence
PROGRESS-Plus framework: place of residence, race, occupation, gender, religion, education, socioeconomic status, social capital, plus personal characteristics/relationships/time-dependent variables
