# Supplementary material for: Factors Affecting Patient and Physician Engagement in Remote Health Care for Heart Failure: Systematic Review
Source: JMIR Cardio. 2022 Apr 6;6(1):e33366. doi: 10.2196/33366 (PMC9021943; doi:10.2196/33366)
Supplement: Multimedia Appendix 1 [file cardio_v6i1e33366_app1.doc]

Multimedia Appendix 1 – Search History

Search History:

2. EMBASE; exp HEART VENTRICLE FUNCTION/; 13980 results.

3. EMBASE; exp HEART FAILURE/; 362955 results.

4. EMBASE; exp HEART EDEMA/; 1172 results.

5. EMBASE; "ventricular failure".ti,ab; 5337 results.

6. EMBASE; "heart failure".ti,ab; 190242 results.

7. EMBASE; "cardiac failure".ti,ab; 13534 results.

8. EMBASE; "ventricular dysfunction".ti,ab; 19906 results.

9. EMBASE; "cardiac insufficiency".ti,ab; 4030 results.

10. EMBASE; 2 OR 3 OR 4 OR 5 OR 6 OR 7 OR 8 OR 9; 412202 results.

11. EMBASE; exp REMOTE SENSING TECHNOLOGY/ OR exp REMOTE CONSULTATION/; 12059 results.

12. EMBASE; ALGORITHMS/ OR exp ARTIFICIAL INTELLIGENCE/ OR AUTOMATIC DATA PROCESSING/ OR CLOUD COMPUTING/ OR exp COMPUTER GRAPHICS/ OR COMPUTER SIMULATION/ OR exp COMPUTER SYSTEMS/ OR exp COMPUTER COMMUNICATION NETWORKS/ OR exp SOFTWARE/ OR exp MOBILE APPLICATIONS/ OR VIDEO GAMES/ OR WEB BROWSER/ OR exp WORD PROCESSING/ OR exp DATA DISPLAY/; 1286363 results.

13. EMBASE; exp PATTERN RECOGNITION, AUTOMATED/ OR exp "NEURAL NETWORKS (COMPUTER)"/ OR exp TELEMEDICINE/ OR exp TELECOMMUNICATIONS/ OR exp TELEVISION/; 97246 results.

14. EMBASE; ("mhealth" OR "telehealth" OR "telemedicine").ti,ab; 11460 results.

15. EMBASE; exp COMPUTER INTERFACE/; 24644 results.

16. EMBASE; exp TEXT MESSAGING/; 2037 results.

17. EMBASE; ((remote OR home OR tele* OR mobile OR internet OR web OR app OR computer OR "smart phone" OR mhealth OR wireless OR "cell phone" OR software OR hardware) adj3 (care OR management OR monitor* OR health OR administration OR consultation OR communication OR medicine OR technolog* OR support OR sens*)).ti,ab; 93082 results.

18. EMBASE; 11 OR 12 OR 13 OR 14 OR 15 OR 16 OR 17; 1383914 results.

19. EMBASE; exp HEALTH BEHAVIOR/ OR exp PATIENT COMPLIANCE/ OR exp MEDICATION ADHERENCE/ OR exp TREATMENT REFUSAL/ OR exp ILLNESS BEHAVIOR/ OR exp INFORMATION SEEKING BEHAVIOR/ OR "INHIBITION (PSYCHOLOGY)"/ OR exp PROACTIVE INHIBITION/ OR exp PERSONAL SATISFACTION/ OR exp HELP-SEEKING BEHAVIOR/ OR exp SELF-CONTROL/ OR exp MOTIVATION/ OR DRIVE/ OR GOALS/ OR INTENTION/ OR PERSONALITY/ OR CHARACTER/ OR INDIVIDUALITY/ OR MOTIVATION/ OR exp LIFE STYLE/ OR exp "BEHAVIORAL DISCIPLINES AND ACTIVITIES"/ OR exp BEHAVIOR CONTROL/; 1124888 results.

20. EMBASE; exp INTERDISCIPLINARY COMMUNICATION/ OR exp PHYSICIAN-NURSE RELATIONS/ OR exp NEGOTIATING/ OR exp PROFESSIONAL-PATIENT RELATIONS/ OR exp NURSE-PATIENT RELATIONS/ OR exp PHYSICIAN-PATIENT RELATIONS/ OR TRUST/ OR MORALE/; 1039099 results.

21. EMBASE; exp "ATTITUDE OF HEALTH PERSONNEL"/ OR exp ATTITUDE TO HEALTH/ OR exp HEALTH KNOWLEDGE, ATTITUDES, PRACTICE/ OR exp "PATIENT ACCEPTANCE OF HEALTH CARE"/ OR exp CULTURALLY COMPETENT CARE/; 501449 results.

22. EMBASE; exp PATIENT PARTICIPATION/ OR exp PATIENT SATISFACTION/ OR exp DECISION MAKING/ OR exp HEALTH KNOWLEDGE, ATTITUDES, PRACTICE/ OR exp HEALTH LITERACY/; 445452 results.

23. EMBASE; "adherence".ti,ab; 112446 results.

24. EMBASE; ((physician OR doctor OR nurse OR "healthcare staff" OR "health care staff" OR patient) adj3 (engagement OR acceptability OR compliance OR concordance OR retention OR enthusiasm OR involvement OR refusal OR negotiation OR participation OR satisfaction OR behaviour OR behavior OR motivation OR intention OR "self-control" OR goal OR goals OR morale OR trust OR attitude OR attitudes OR drive OR personality OR character OR individuality OR "life style")).ti,ab; 84951 results.

25. EMBASE; 19 OR 20 OR 21 OR 22 OR 23 OR 24; 2112225 results.

26. EMBASE; 10 AND 18; 16662 results.

27. EMBASE; 25 AND 26; 2540 results.
