# Supplementary material for: Factors Affecting Patient and Physician Engagement in Remote Health Care for Heart Failure: Systematic Review
Source: JMIR Cardio. 2022 Apr 6;6(1):e33366. doi: 10.2196/33366 (PMC9021943; doi:10.2196/33366)
Supplement: Multimedia Appendix 3 [file cardio_v6i1e33366_app3.docx]

Multimedia Appendix 3. Characteristics of studies included with the first order codes generated from each study

| Study | Intervention | Methods and sampling | Codes generated |
| --- | --- | --- | --- |
| Abidi 2013 [51]: Usability Evaluation of Family Physicians’ Interaction with the Comorbidity Ontological Modeling and ExecuTion System (COMET)  Aim: Assessing usability of intervention with family physicians  Country: Canada  Funding: Green Shield Canada Foundation | Name: Comorbidity Ontological Modeling and ExecuTion System (COMET)  Type: Clinical Decision Tool  Setting: Physician workplace  Duration: 1 day  Details: 15-minute training session involving clinical scenarios to simulate patients with various HF issues and diagnostic problems. | Sample: 10 physicians  Sampling: Purposive  Data: Observation, questionnaires, ‘Think aloud’ sessions  Analysis: Grounded theory  Quality score: + | Healthcare staff –ve experiences:  *“Inflexible guidance”*  *“Lack of training”*  *“Lack of education”*  *“Not user friendly”* |
| Agrell 2000 [18]: Patients’ Perceptions Regarding Home Telecare  Aims: To elicit patients’ perceptions regarding home telecare  Countries: USA & Mexico  Funding: Not reported | Name: American TeleCare  Type: Remote monitoring (Telemonitoring)  Setting: Patient’s home  Duration: Not stated  Details: Telecare in patient’s homes | Sample: 15 patients  Sampling: Convenience  Data: Interviews, questionnaires  Analysis: Not stated  Quality score: + | Patient +ve experiences:  *“Patient-staff communication”*  *“feels looked after”*  Patient -ve:  *“Lack of patient-staff communication”*  *“Not user friendly”*  *“Lack of privacy/ security”* |
| Akyar 2019 [58]: Using Patients and Their Caregivers Feedback to Develop ENABLE CHF-PC: An Early Palliative Care Intervention for Advanced Heart Failure  Aim: Evaluate the intervention from patient perspectives  Country: USA  Funding: Dartmouth SYNERGY Center for Clinical and Translational Science | Name: ENABLE Comprehensive Heartcare For Patient and Caregivers  Type: Educational tool and online management  Setting: Patient’s home  Duration: 6 weeks  Details: An in-person palliative care assessment followed by weekly telehealth coaching sessions with a specialist nurse on palliative care topics, and monthly follow-up | Sample: 8 patients, 8 carers  Sampling: Purposive  Data: Interviews, questionnaires  Analysis: Content analysis  Quality score: ++ | Patient +ve experiences:  *“Psychosocial support”*  *“Pt-staff communication”*  *“feels looked after”*  *“Clinical knowledge”*  *“Confidence and motivation in self-care”*  *“Flexible”*  Patient –ve experiences:  *“Lack of education”*  *Lack of options*  Carer +ve experiences:  *Feel patient is ‘looked after’*  *Extra support*  *Informative/Educational*  *User friendly*  Carer –ve experiences:  *Extra responsibility*  *Lack of education* |
| Barron 2013 [54]: Feasibility of providing personalized health information to older adults and their caregivers  Aim: Assess feasibility of intervention  Country: USA  Funding: Not reported | Name: Johns Hopkins Patient Portal  Type: Patient health information platform  Setting: Clinic  Duration: 1 day  Details: Internet-based patient portal to help family caregivers to partner more effectively with the health care team. | Sample: 23 patients  Sampling: Convenience  Data: Interviews, questionnaires  Analysis: Not stated  Quality score: - | Patient +ve experiences:  *“User friendly”*  Patient –ve experiences:  *“Not user friendly”*  *“Lack of training”*  *“Lack of education”*  Carer +ve experiences:  *“Extra support”*  *“Informative/ Educational”*  *“User friendly”*  Carer –ve experiences:  *“Lack of education”*  *“Not user friendly”* |
| Bartlett 2014 [19]: The SMART personalised self-management system for congestive heart failure: results of a realist evaluation  Aims: A realist evaluation of the intervention  Country: UK  Funding: Engineering and Physical Sciences Research Council | Name: SMART Personalised Self-Management System (PSMS) for heart failure  Type: Remote monitoring (Telemonitoring) and self-monitoring  Setting: Patient’s home  Duration: 1-3 months  Details: A touch screen computer and a touch screen mobile device, as well as sensor devices to measure weight and blood pressure were provided. Together, the components of the system encouraged goal setting, enabled self-monitoring of symptoms and behaviour, encouraged the ongoing review of progress towards goals and provided access to quality-assured information about the condition. | Sample: 7 patients  Sampling: Not stated  Data: Interviews  Analysis: Realist evaluation  Quality score: + | Patient +ve experiences:  *“Feels looked after”*  *“Clinical knowledge”*  *“Confidence/ motivation in self-care”*  *“Involvement in self-care”*  *“Confidence in management decisions”*  *“Improvement from usual care”*  *“User friendly”*  *“New technology”*  Patient –ve experiences:  *“Lack of feedback”*  *“No effect on self-care”*  *“Lack of improvement”*  *“Technical difficulties”*  *“Lack of portability”*  *“Technology overwhelming”*  *“Irrelevant training”*  *“Intrusive”*  *“Cost”*  *“Lack of options”*  *“Extra work = tiring”* |
| Bekelman 2014 [64]: Feasibility and Acceptability of a Collaborative Care Intervention To Improve Symptoms and Quality of Life in Chronic Heart Failure: Mixed Methods Pilot Trial  Aim: To determine the feasibility and acceptability of the intervention and identify necessary improvements  Country: USA  Funding: Department of Veterans Affairs | Name: The CASA (Collaborative Care to Alleviate Symptoms and Adjust to Illness)  Type: Telephone consultations  Setting: Patient’s home  Duration: 3-6 months  Details: 3 step intervention: 1) nurse phone visits involving structured symptom assessments to alleviate breathlessness, fatigue, pain, or depression; 2) structured phone counselling targeting adjustment to illness and depression; 3) weekly team meetings with a palliative care specialist, cardiologist, and primary care physician focused on medical recommendations to primary care providers to improve symptoms. | Sample: 17 patients  Sampling: Not stated  Data: Interviews  Analysis: Not stated  Quality score: + | Patient +ve experiences:  *“Psychosocial support”*  *“Patient-staff communication”*  *“Feels looked after”*  *“Clinical knowledge”*  *“Confidence/ motivation in self-care”*  *“Involvement in self-care”*  *“User friendly”*  Patient –ve experiences:  *“Reliance on staff”*  *“Lack of patient-staff communication”*  *“Lack of improvement”*  *“Not user friendly”*  *“Lack of education”*  *“Irrelevant training”*  *“Lack of options”* |
| Cajita 2018 [46]: Facilitators of and Barriers to mHealth Adoption in Older Adults With Heart Failure  Aim: To assess the perceptions of older adults with heart failure regarding the use of mobile technology and to identify potential facilitators of and barriers to mHealth adoption.  Country: USA  Funding: National Institute of Nursing Research, Council for the Advancement of Nursing Science–Southern Nursing Research Society, and Heart Failure Society of America. | Name: ‘mHealth’  Type: Remote monitoring (Telemonitoring), Self-monitoring  Setting: Hospital  Duration: One day  Details: Quantitative survey and interview about mobile technology, followed by a video of a mHealth monitoring system and a health-related app and further follow-up questions. The video showed a typical monitoring system composed of a weighing scale, blood pressure cuff, and pulse oximeter, all with wireless connections to a mobile device. Finally, the participants were presented with an app that is designed to track dietary salt  intake. After a demonstration by the research assistant, participants were asked to use the app while sharing their thoughts. | Sample: 10 participants  Sampling: Purposive  Data: Interviews  Analysis: Thematic and content analysis  Quality score: ++ | Patient +ve experiences:  *“Psychosocial support”*  *“Patient-staff communication”*  *“Feels looked after”*  *“Clinical knowledge”*  *“Confidence/ motivation in self-care”*  *“Flexible”*  Patient –ve experiences:  *“Lack of Education”*  *“Lack of options”*  Carer +ve experiences:  *“Feel patient is looked after”*  *“Extra support”*  *“Informative/ educational”*  *“User friendly”*  Carer –ve experiences:  *“Extra responsibility”*  *“Lack of education”* |
| Chantler 2016 [68]: Creating connections - the development of a mobile-health monitoring system for heart failure: Qualitative findings from a usability cohort study  Aim: Learning user capacity and preferences from interactions with a mobile-health monitoring system  Country: UK  Funding: National Institute for Health Research (NIHR) Oxford BRC programme, and by an NIHR Career Development Fellowship | Name: ‘SUPPORT HF mhealth monitoring system’  Type: Self-monitoring, Remote monitoring (Telemonitoring)  Setting: Patient’s home  Duration: 3-4 months  Details: Mobile application on an Android-based tablet computer connected to a blood pressure monitor and a set of weighing scales using the Bluetooth. Participants were asked to complete symptom diaries and take measurements on a regular basis, and complete quality of life questionnaires every three months. These data were transmitted to the study team to review the participants’ health status remotely. In-built alerts were issued if a participant’s measurements indicated any deterioration in their condition that required them to contact their general practitioner (GP), and the study team could also send text messages to comment on participants’ health status. The application also included features that allowed participants to review their personal readings via a graphical display, access educational materials and communicate with the study team. | Sample: 58 patients  Sampling: Purposive  Data: Interviews  Analysis: Ethnography, Thematic and Framework analysis  Quality score: ++ | Patient +ve experiences:  *“Psychosocial support”*  *“Patient-staff communication”*  *“Feels looked after”*  *“Clinical knowledge”*  *“Confidence/ motivation in self-care”*  *“Involvement in self-care”*  *“Confidence in management decisions”*  *“Improvement from usual care”*  *“User friendly”*  *“New technology”*  Patient –ve experiences:  *“Responsibility = anxiety”*  *“No effect on self-care”*  *“Not user friendly”*  *“Technology overwhelming”*  *“Lack of training”*  *“Irrelevant training”*  *Cannot replace hospital care”*  *“Technology not needed”* |
| Dang 2017 [69]: Mobile Phone Intervention for Heart Failure in a Minority Urban County Hospital Population: Usability and Patient Perspectives  Aim: To pilot the telemonitoring system and determine its impact on self-efficacy, knowledge, and quality of life  Country: USA  Funding: Spar State of Florida Department of Health’s James and Esther King Biomedical Re- search Program | Name: ‘Mobile Phone Intervention’  Type: Self-monitoring, Remote monitoring (Telemonitoring)  Setting: Patient’s home  Duration: 3 months  Details: The telemonitoring system required the participants to answer 10 daily questions regarding their weight and HF symptoms via a mobile phone. Participants received 3 messages 15 min apart if they did not respond to the first automated message. If responses indicated possible HF worsening, the patient received a message asking him/her to contact the study co-ordinator who was able to view the data and received an alert on his/her study mobile phone. The study coordinator then coordinated the patients’ care with the providers in the Heart Failure Clinic and acted as the intermediary between the patients and the providers. | Sample: 42 patients  Sampling: Not stated  Data: Interviews  Analysis: Content analysis  Quality score: - | Patient +ve experiences:  *“Feels looked after”*  *“Clinical knowledge”*  *“Confidence/ motivation in self-care”*  *“Involvement in self-care”*  *“Improvement from usual care”*  *“User friendly”*  *“New technology”*  *“Flexible”*  Patient –ve experiences:  *“Lack of pt-staff communication”*  *“Technical difficulties”*  *“Not user friendly”*  *“Intrusive”*  *“Cost”*  *“Lack of options”*  *“Lack of efficiency/ co-ordination”* |
| Dinesen 2008 [20]: Under surveillance, yet looked after: Telehomecare as viewed by patients and their spouse/partners  Aim: To understand the experiences and attitudes of patients and their spouses/partners with regard to the application of telehomecare technology within home hospitalisation  Country: Denmark  Funding: Spar Nord Fonden, Det Obelske Familiefond, Jyske Bank and Aalborg University | Name: ‘Home hospitalisation’  Type: Remote monitoring (Telemonitoring)  Setting: Patient’s home  Duration: up to 1 week  Details: District nurse visits twice daily - morning and evening and phone hospital nurses to exchange information and jobs. DN collects data: BP, pulse, weight, INR, ECG and sends wirelessly to hospital. Information is used for twice daily ward round. Patient discharged when stable. | Sample: 8 patients, 6 spouses  Sampling: Purposive  Data: Interviews  Analysis: Thematic analysis  Quality score: ++ | Patient +ve experiences:  *“Patient-patient communication”*  *“Patient-staff communication”*  *“Feels looked after”*  *“Clinical knowledge”*  *“Confidence/ motivation in self-care”*  *“Involvement in self-care”*  *“Confidence in management decisions”*  *“Improvement from usual care”*  *“New technology”*  *“Flexible”*  *“Comfort/ freedom at home”*  Patient –ve experiences:  *“Technical difficulties”*  *“Adds extra concern for carers”*  Carer +ve experiences:  *“Feel patient is looked after”*  *“Extra support”*  Carer –ve experiences:  *“Extra responsibility”*  *“Change is stressful”*  *“Causes concern for patient”*  *“Control taken away”*  *“Invasion of privacy”*  *“Lack of education”*  *“Lack of improvement”*  *“Technical difficulties = stressful”* |
| Dubois 2001 [21]: Evaluation of patients’ satisfaction with hospital-at-home care  Aim: To measure patients’ satisfaction with the intervention  Country: Switzerland  Funding: Public Health Department of the Canton of Vaud | Name: The H-Hcare Program  Type: Remote monitoring (Telemonitoring) and Home visits  Setting: Patient’s home  Duration: 1-3 months  Details: A home-care program consisting of regularly scheduled nurse visits for medical patients already in hospital to be discharged home with rather than staying in hospital. | Sample: 107 patients  Sampling: Not stated  Data: Interviews, questionnaires  Analysis: Thematic analysis  Quality score: + | Patient +ve:  *“Psychosocial support”*  *“Feels looked after”*  *“Confidence in management decisions”*  *“Saves travel time”*  *“Flexible”*  *“Comfort/ Freedom at home”*  Patient –ve experiences:  *“Lack of patient-staff communication”*  *“Responsibility = anxiety”*  *“Unpredictable clinical management”*  *“Lack of improvement”*  *“Technical difficulties”*  *“Lack of training”*  *“Cost”*  *“Lack of privacy/ security”*  *“Lack of options”*  *“Adds extra concern for carers”*  *“Lack of support for care”*  *“Extra work = tiring”*  *“Uncomfortable at home”*  *“Lack of efficiency/ co-ordination”*  *“Cannot replace hospital care”*  Carer +ve experiences:  *“Feel patient is looked after“*  Carer –ve experiences:  *“Extra responsibility”*  *“Change is stressful”* |
| Earnest 2004 [55]: Use of a Patient-Accessible Electronic Medical Record in a Practice for Congestive Heart Failure: Patient and Physician Experiences  Aim: To evaluate the experiences of patients and physicians in a clinical trial of the intervention  Country: USA  Funding: Commonwealth Fund | Name: SPPARO, System Providing Patients Access to Records Online  Type: Patient health information platform  Setting: Patients’ home and clinic  Duration: 1-2 years  Details: Patient-accessible electronic medical record that provides access to clinical notes, test results, health information, and a method of sending and receiving electronic messages to and from the clinic staff. | Sample: 16 patients, 7 physicians  Sampling: Not stated  Data collection: Focus-groups, interviews  Analysis: Grounded theory  Quality score: ++ | Patient +ve experiences:  *“Patient-staff communication”*  *“Feels looked after”*  *“Clinical knowledge”*  *“Follow up/ co-ordination of care”*  *“Confidence/ motivation in self-care”*  *“Involvement in self- care”*  *“Confidence in management decisions”*  *“Popular”*  Patient –ve experiences:  *“Lack of patient-staff communication”*  *“Medical jargon”*  *“Not user friendly”*  *“Lack of training”*  *“Lack of education*  *“Lack of privacy/ security”*  Healthcare staff +ve experiences:  *“Familiarity/ communication with patient”*  *“Reduces error”*  *“Important”*  *“Encourages patient self-care”*  *“Increases patient knowledge”*  *“No change in workload”*  Healthcare staff –ve experiences:  *“Increases patient concern/ confusion”*  *“Lack of patient-staff communication”*  *“No improvement”*  *“Increases errors”*  *“Cost”*  *“Health inequality”* |
| Fairbrother 2014 [22]: Telemonitoring for chronic heart failure: the views of patients and healthcare professionals – a qualitative study  Aim: To understand the views of patients and professionals on the acceptability and perceived usefulness of telemonitoring in the management of chronic heart failure.  Country: Scotland  Funding: Scottish Centre for Telehealth and Telecare | Name: Intel Health Guide (IHG)  Type: Remote monitoring (Telemonitoring)  Setting: Patient’s home  Duration: 3-6 months  Details: Touch-screen device allowing a daily self-assessment of symptoms using an online questionnaire. The device was connected to sensors that measured pulse rate, oxygen saturation, blood pressure, and weight daily. The IHG also contained educational content (in the form of online video-based content) to support patient self-management. | Sample: 18 patients  Sampling: Purposive  Data: Interviews  Analysis: Framework analysis  Quality score: ++ | Patient +ve experiences:  *“Patient-staff communication”*  *“Feels looked after”*  *“Clinical knowledge”*  *“Confidence/ motivation in self-care”*  *“Involvement in self-care”*  *“User friendly”*  *“Popular”*  Patient –ve experiences:  *“Reliance on staff”*  *“Lack of patient-staff communication”*  *“Responsibility = anxiety”*  *“Unpredictable clinical management”*  *“No effect on self-care”*  *“Lack of improvement”*  *“Technical difficulties”*  *“Intrusive”*  *“Cost”*  Healthcare staff +ve experiences:  *“Familiarity/ communication with patient”*  *“Better knowledge of patient status”*  *“Pro-active management”*  *“Confidence in management”*  Healthcare staff –ve experiences:  *“Perceived dependence on staff”*  *“Lack of patient-staff communication”*  *“Not linked to records”*  *“Inflexible guidance”*  *“Not user friendly”*  *“Lack of education”*  *“No change in patient self-care”*  *“No improvement”*  *“Increased workload”*  *“Patient selection decisions”*  *“Cost”* |
| Finkelstein 2011 [23]: Implementing Home Telemanagement of Congestive Heart Failure Using Xbox Gaming Platform  Aim: to explore whether it is possible to take advantage of the simplicity, popularity, and low cost of the Xbox360 to build a platform able to deliver a comprehensive disease management program within patients’ homes  Country: USA  Funding: Not stated | Name: Home Automated Telemanagement (HAT) system  Type: self-monitoring, remote monitoring (telemonitoring), clinical decision tool, patient health information platform, online management, educational tool  Setting: Patient’s home  Duration: Not stated  Details: The HAT system consists of home unit, HAT server and clinician unit. The home unit uses an Xbox 360 gaming platform and the Xbox360 controller for input. It allows a patient to record symptoms and weight and gives feedback about their condition over time. The physician can access the CHF HAT website to review patient data, track progress, make changes to medications, and set alerts. The HAT server then uses clinical decision support algorithms which alert the physician if any ominous trend is detected. | Sample: 10 patients  Sampling: Not stated  Data: Interviews  Analysis: Not stated  Quality score: - | Patient +ve experiences:  *“Feels looked after”*  *“Clinical knowledge”*  *“Confidence/ motivation in self-care”*  *“User friendly”*  *“Popular”*  *“Flexible”*  Patient –ve experiences:  *“Not user friendly”* |
| Grace 2017 [47]: Perceptions of seniors with heart failure regarding autonomous zero-effort monitoring of physiological parameters in the smart-home environment  Aim: To understand the perceptions of seniors with heart failure regarding smart-home systems to monitor their physiological parameters  Country: Canada  Funding: Canadian Institutes of Health Research | Name: Smart-home system  Type: Remote monitoring (Telemonitoring), and self-monitoring  Setting: Patient’s home  Duration: 1 day  Details: The smart-home was crafted by embedding passive sensors into commonly-found objects and furniture: (1) A network of 16 accelerometers installed on a blanket (used in the bedroom) to capture chest motion and calculate respiration; (2) Capacitively-coupled (CC) electrodes and load cells installed on a chair (used in the dining area) to record electrocardiogram (ECG) and ballistocardiogram (BCG), which can be used to measure heart rate and blood pressure; (3) Load cells employed under the legs of the bed to measure body weight; (4) Infrared thermometry for non- contact body temperature recording from a person’s face while he or she is watching TV; and (5) A custom-built ﬂoor tile which contained embedded dry electrodes and load cells to measure ECG and BCG signals. | Sample: 26 patients  Sampling: Purposive  Data: Interviews  Analysis: Content analysis  Quality score: ++ | Patient +ve experiences:  *“Feels looked after”*  *“Clinical knowledge”*  *“Follow up / co-ordination of care”*  *“User friendly”*  *“Comfort / freedom at home”*  Patient –ve experiences:  *“Responsibility = anxiety”*  *“Technical difficulties”*  *“Not user friendly”*  *“Technology overwhelming”*  *“Intrusive”*  *“Lack of privacy / security”*  *“Lack of options”*  *“Technology not needed”* |
| Green 2006 [52]: Information system support as a critical success factor for chronic disease management: Necessary but not sufﬁcient  Aim: To identify critical success factors enabling clinical knowledge about effective and efﬁcient chronic care management to be translated into primary care practice  Country: Canada  Funding: Canadian Institute for Health Research | Name: Chronic disease management (CDM) collaborative  Type: Clinical decision tool  Setting: Clinic  Duration: 3-5 years  Details: A web-based patient registry system with guideline-based ﬂow sheet that allowed physicians to identify the patients in their practice with target chronic diseases and to monitor their care against a standard. | Sample: 30 physicians  Sampling: Not stated  Data: Interviews  Analysis: Critical success factor analysis  Quality score: + | Healthcare +ve experiences:  *“Encourages teamwork”*  *“Better knowledge of patient status”*  *“Confidence in management”*  *“Identifies priorities in care”*  *“Reduces error”*  *“Staff education”*  *“User friendly”*  *“Technical support”*  *“Security/ privacy”*  *“No change in workload”*  *“Incentivisation”* |
| Gund 2008 [24]: Design Evaluation of a Home-Based Telecare System for Chronic Heart Failure Patients  Aim: Evaluation of the usability of the intervention with heart failure patients.  Country: Sweden  Funding: VINNOVA | Name: Care@Distance  Type: Remote monitoring (Telemonitoring), and self-monitoring  Setting: Patient’s home  Duration: 1-3 months  Details: Three component telecare system consisting of a home based patient terminal, an internet networked database, and a web portal for the care providers. The home terminal is a tablet PC that will record blood pressure, weight, and symptoms via a blood pressure monitor and scale. Data from the patient terminal is accessed by the health care providers through the web portal, which is also used for managing the home terminal. | Sample: 2 patients  Sampling: Convenience  Data: Interviews  Analysis: Not stated  Quality score: - | Patient +ve experiences:  *“Patient-staff communication”*  *“Feels looked after”*  *“User friendly”*  *“Popular”*  *“Saves travel time”*  *“Flexible”*  Patient –ve experiences:  *“Technical difficulties”*  *“Not user friendly”*  *“Intrusive”*  *“Lack of options”*  Healthcare staff –ve experiences:  *“Not user friendly”*  *“Lack of options”* |
| Hall 2014 [41]: Heart Failure Patients’ Perceptions and Use of Technology to Manage Disease Symptoms  Aim: This study explores patients’ perceptions and current use of technology for managing HF symptoms  Country: USA  Funding: Finger Fellowship in Sustainable Health | Name: Blue Scale (Blue Box, Inc., Houston, TX)  Type: Remote monitoring (Telemonitoring)  Setting: Patient’s home  Duration: Not stated  Details: The Blue Scale device tracks multiple vital sign readings important for HF patient home monitoring while the patient grasps handles that are large and attached to a reinforced base for adequate support and fall prevention. | Sample: 15 patients  Sampling: Not stated  Data: Interviews  Analysis: Constant comparison  Quality score: + | Patient +ve experiences:  *“Patient-staff communication”*  *“Feels looked after”*  *“Clinical knowledge”*  *“Confidence/ motivation in self-care”*  *“Improvement from usual care”*  *“New technology”*  *“Saves travel time”*  *“Comfort/ freedom at home”*  Patient –ve experiences:  *“Reliance on staff”*  *“Technical difficulties”*  *“Medical jargon”*  *“Technology overwhelming”*  *“Lack of education”*  *“Unreliable information”*  *“Cost”*  *“Lack of privacy/ security”*  *“Extra work = tiring”* |
| Heisler 2007 [65]: “I Am Not Alone”: The Feasibility and Acceptability of Interactive Voice Response–Facilitated Telephone Peer Support Among Older Adults With Heart Failure  Aim: Evaluating the feasibility and acceptability of the intervention to facilitate telephone peer support among older adults with heart failure  Country: USA  Funding: Not stated | Name: Interactive voice-response based platform  Type: Peer-support system  Setting: Patient’s home  Duration: 1-3 months  Details: Peer support in conjunction with a structured program of education and assistance. Participants were asked to contact their partner at least once a week using the toll-free IVR calling line. | Sample: 20 patients  Sampling: Convenience  Data: Interviews, questionnaires  Analysis: Not stated  Quality score: + | Patient +ve experiences:  *“Psychosocial support”*  *“Patient-patient communication”*  *“Confidence/ motivation in self-care”*  Patient –ve experiences:  *“Lack of patient-patient communication”*  *“Lack of patient-staff communication”*  *“No effect on self-care”*  *“Lack of improvement”*  *“Lack of training”* |
| Heckemann 2016 [48]: Discovering untapped relationship potential with patients in telehealth: a qualitative interview study  Aim: To explore factors that influence relationship building between telehealth professionals and patients with chronic illness over a distance, from a telehealth professional's perspective.  Country: Germany  Funding: Gesellschaft für Patientenhilfe DGB mbh (GPH) | Name: Unstated  Type: Remote monitoring (Telemonitoring)  Setting: Patient’s home  Duration: months to years  Details: Patients report daily on signs, symptoms and body weight. These data are automatically transferred to the centre. Telephone calls are the exclusive means of communication. During the initial phone call, the THPs assess factors such as the patient’s mobility, nutrition, social circumstances and cognitive abilities. Scheduled support calls, that also include teaching units, then occur every 4–6 weeks, unless the patient’s condition necessitates intervention. | Sample: 20 telehealth professionals  Sampling: Not stated  Data: Focus groups  Analysis: Content analysis  Quality score: ++ | Healthcare staff +ve experiences:  *“Familiarity / communication with patient"*  *“Encourages patient self-care”*  *“Better knowledge of patient status”*  *“Important”*  *“Improvement from usual care”*  *“Increases patient knowledge”*  *“Security / Privacy”*  Healthcare staff –ve experiences:  *“Perceived dependence on staff”*  *“Lack of patient-staff communication”* |
| Hunting 2015 [25]: A multi-level qualitative analysis of Telehomecare in Ontario: challenges and opportunities  Aim: To explore the factors which facilitate or impede the implementation of the program  Country: Canada  Funding: Ontario Ministry of Health and Long Term Care (MOHLTC) | Name: Ontario Telemedicine Network (OTN)  Type: Remote monitoring (Telemonitoring)  Setting: Patient’s home  Duration: 6 months  Details: The program involves: i) telehomecare nurses with whom patients can interact by telephone; ii) daily transmission of patient data (weight, blood pressure, oxygen levels, and answers to daily questionnaire) to a nurse via a remote monitoring device; iii) individualized care based on patient needs (e.g., following up with patient if their data is outside of a normal range and weekly coaching sessions); and iv) communication regarding patient health concerns between the nurse and other members of the patient’s circle of care. | Sample: 39 patients/ caregivers, 23 healthcare staff, 2 technicians, 12 administrators, 13 decision makers  Sampling: Purposive  Data: Interviews, ethnographic observation  Analysis: Grounded theory thematic analysis  Quality score: ++ | Patient +ve experiences:  *“User friendly”*  *“Confidence/ motivation in self-care”*  *“Feels looked after”*  *“Saves travel time”*  *“Patient-staff communication”*  *“Follow up/ co-ordination of care”*  Patient –ve experiences:  *“Not user friendly”*  *“Language barriers”*  *“Reliance on staff”*  *“Intrusive”*  *“Lack of portability”*  *“No effect on self-care”*  *“Irrelevant training”*  Carer +ve experiences:  *“Feels patient is looked after”*  Healthcare staff +ve experiences:  *“Encourages patient self-care”*  *“Saves travel time”*  *“Technical support”*  *“Encourages teamwork”*  *“Better knowledge of patient status”*  *“Automated saves time”*  *“Important”*  *“Local champions”*  *“Improvement from usual care”*  Healthcare staff –ve experiences:  *“Increased workload”*  *“Perceived dependence on staff”*  *“Not linked to records”*  *“Lack of staff-staff communication”*  *“Lack of patient-staff communication”*  *“Patient selection decisions”*  *“Health inequality”*  *“Slow to change practice”*  *“Cost”* |
| Johnston 2010 [26]: Automated weight monitoring in chronic heart failure: the excluded majority  Aim: To Investigate how the intervention might affect the care of the patients in a wider sense than solely the cost-effectiveness  Country: UK  Funding: Not stated | Name: Remote automated weight monitoring system  Type: Remote monitoring (Telemonitoring)  Setting: Patient’s home  Duration: Not stated  Details: Not stated | Sample: 14 patients, 10 carers, 4 nurses  Sampling: Not stated  Data: Focus groups, interviews  Analysis: Thematic analysis  Quality score: - | Patient +ve experiences:  *“Psychosocial support”*  *“Feels looked after”*  *“Confidence/ motivation in self-care”*  *­*Patient –ve experiences:  *“Lack of patient-staff communication”*  *“Lack of feedback”*  *“Lack of improvement”*  *“Technical difficulties”*  *“Not user friendly”*  *“Lack of education”*  *“Intrusive”*  *“Cost”*  Carer +ve experiences:  *“Feels patient is looked after”* |
| Kenealy 2015 [27]: Telecare for Diabetes, CHF or COPD: Effect on Quality of Life, Hospital Use and Costs. A Randomised Controlled Trial and Qualitative Evaluation  Aim: To assess the effect of telecare on health related quality of life, self-care, hospital use, costs and the experiences of patients, informal carers and health care professionals  Country: New Zealand  Funding: Primary Health Care Strategic Innovations Fund | Name: ‘Health Hub ’ supplied by Docobo  Type: Remote monitoring (Telemonitoring) and self-monitoring  Setting: Patient’s home  Duration: 6-12 months  Details: The hub was a small device with a LCD display to provide instructions, ask disease-specific questions, or convey short messages from the nurses monitoring the data. Patients were also provided with electronic weighing scales, a blood pressure monitor, a pulse oximeter and a glucometer for diabetics. Patients enter data manually into the hub which is then relayed to the monitoring stations where they were viewed by the nurses. | Sample: 47, combined patients, carers and healthcare professionals  Sampling: Purposive  Data: Focus groups, interviews  Analysis: Thematic analysis  Quality score: ++ | Patient +ve experiences:  *“Psychosocial support”*  *“Patient-staff communication”*  *“Feels looked after”*  *“Clinical knowledge”*  *“Confidence/ motivation in self-care”*  *“Involvement of self-care”*  *“Confidence in management decisions”*  *“Improvement from usual care”*  *“New technology”*  *“Popular”*  *“Saves travel time”*  *“Flexible”*  Patient –ve experiences:  *“Technical difficulties”*  *“Lack of portability”*  *“Extra work = tiring”*  Carer +ve experiences:  *“Feels patient is looked after”*  *“Informative/ educational”*  Healthcare staff +ve experiences:  *“Familiarity/ communication with patient”*  *“Important”*  *“Encourages patient self-care”*  *“Increases patient knowledge”*  *“No change in workload”*  Healthcare staff –ve experiences:  *“Technical difficulties”*  *“Increased workload”*  *“Increases errors”* |
| Leslie 2006 [53]: Clinical decision support software for management of chronic heart failure: Development and evaluation  Aim: To develop and evaluate clinical decision support software (CDSS) to aid physicians treat patients with chronic heart failure (CHF)  Country: UK  Funding: Not stated | Name: Clinical decision support software (CDSS)  Type: Clinical decision tool  Setting: Clinic and hospital  Duration: Not stated  Details: The evidence base used to generate the clinical information within the tool was derived from published guidelines and papers. This information was used to create a series of ‘yes/no’ ﬂow diagrams for diagnosis, initiation and up-titration of drug treatments. Various combinations of these ‘inputs’ produced a range of ‘outputs’ designed as short phrases containing clinical advice to allow users to check their suspicions against a pattern of clinical features which have been shown to exclude or include a diagnosis of heart failure, e.g. a normal electrocardiogram, history of previous myocardial infarction etc. | Sample: 5 healthcare staff  Sampling: Not stated  Data: Interview  Analysis: Not stated  Quality score: - | Healthcare staff +ve experiences:  *“Confidence in management”*  *“Identifies priorities in care”*  *“Improvement from usual care”*  *“Important”*  *“Increases patient knowledge”*  *“Staff education”*  *“User friendly”*  *“Technical support”*  *“Automated saves time”*  Healthcare staff –ve experiences:  *“Inflexible guidance”*  *“Not user friendly”*  *“Computer literacy”*  *“Lack of equipment/ support”*  *“Lack of education”*  *“Increased workload”*  *“Slow to change practice”*  *“Lack of options”* |
| Lind 2014 [28]: Telehealth for “the Digital Illiterate” – Elderly Heart Failure Patients’ Experiences  Aim: The aim of this paper was to explore and describe the patients’ and spouses’ experiences in using the system  Country: Sweden  Funding: European Regional Development Fund | Name: Digital pen technology (www.anoto.com),The Health Diary system  Type: Remote monitoring (Telemonitoring) and self-monitoring  Setting: Patient’s home  Duration: 1-2 years  Details: This comprises the pen and ordinary paper with a printed close-to-invisible pattern read by a camera inside the digital pen. The pen is used as an ordinary ball-point pen, but the strokes made by the pen are recorded and transferred via mobile internet to a server. The Health Diary system supported daily reports on shortness of breath, intake of medications, weight and other measurements. The diary also allowed free text messages to the care provider. The system was monitored by health professionals and generated alarms if patient-reported values were below/above certain limits. | Sample: 7 patients  Sampling: Not stated  Data: Interviews  Analysis: Content analysis  Quality score: + | Patient +ve experiences:  *“Patient-staff communication”*  *“Feels looked after”*  *“Clinical knowledge”*  *“Involvement in self-care”*  *“Confidence in management decisions”*  *“Improvement from usual care”*  *“User friendly”*  *“Saves travel time*  Patient –ve experiences:  *“Lack of improvement”*  *“Not user friendly”*  *“Technology overwhelming”* |
| Lowrie 2014 [66]: Experiences of a community pharmacy service to support adherence and self-management in chronic heart failure  Aim: We aimed to explore and portray in detail, the perspectives of patients receiving, and pharmacists delivering an enhanced, pay for performance community pharmacy HF service  Country: UK  Funding: Not stated | Name: Community pharmacy-based heart service  Type: Pharmacy consultations  Setting: Pharmacy  Duration: Not stated  Details: Training received by each participating community pharmacist comprised 3 h of contact time with experienced pharmacists and nurses, including mock consultations with patients. The community pharmacy was then expected to deliver the service to referred patients on a monthly or two–monthly basis. The content of each consultation included review of each medicine, monitoring of symptoms, identified care issues, adherence issues, weight monitoring, smoking status, and referral. | Sample: 65 patients, 10 pharmacists  Sampling:  Data: Focus groups, interviews  Analysis: Framework analysis  Quality score: + | Patient +ve experiences:  *“Psychosocial support”*  *“Patient-staff communication”*  *“Feels looked after”*  *“Clinical knowledge”*  *“Confidence/ motivation in self-care”*  *“Involvement in self-care”*  *“Improvement from usual care”*  *“Popular”*  *“Saves travel time”*  Patient –ve experiences:  *“Lack of improvement”*  *“Lack of education”*  *“Lack of efficiency/ co-ordination”*  Healthcare staff +ve experiences:  *“Familiarity/ communication with patient”*  *“Better knowledge of patient status”*  *“Pro-active management”*  *“Confidence in management”*  *“Important”*  *“Encourages patient self-care”*  *“Increases patient knowledge”*  *“User friendly”*  Healthcare staff –ve experiences:  *“Lack of patient-staff communication”*  *“Lack of staff-staff communication”*  *“Inflexible guidance”*  *“Lack of training”*  *“Lack of education”*  *“No change in patient self-care”*  *“Increased workload”* |
| Lundgren 2015 [56]: Internet-based cognitive behaviour therapy for patients with heart failure and depressive symptoms: A proof of concept study  Aim: To evaluate the feasibility of the ICBT program in regard to depressive symptoms, the time used by health care providers to give feedback, and participants’ perceptions of the ICBT program  Country: Sweden  Funding: Medical Research Council of Southeast Sweden | Name: Internet-based-CBT (ICBT)  Type: Patient health information platform and online management  Setting: Patient’s home  Duration: 1-3 months  Details: The treatment program and feedback were delivered via an internet platform. The core of the program material was adapted to heart failure patients and included psycho-education, behaviour activation, and problem-solving over a nine-week program. | Sample: 7 patients  Sampling: Not stated  Data: Interviews  Analysis: Content analysis  Quality score: + | Patient +ve experiences:  *“Clinical knowledge”*  *“Confidence/ motivation in self-care”*  *“Flexible”*  Patient –ve experiences:  *“Technical difficulties”*  *“Extra work = tiring”* |
| Lundgren 2018 [59]: Patient Experiences of Web-Based Cognitive Behavioral Therapy for Heart Failure and Depression: Qualitative Study  Aim: To explore and describe the experiences of participating and receiving health care through a wCBT intervention among persons with heart failure and depressive symptoms.  Country: Sweden  Funding: Medical Research Council of Southeast Sweden | Name: Web-based CBT (wCBT)  Type: Patient health information platform and online management  Setting: Patient’s home  Duration: 1-12 months  Details: The wCBT program consists of text and assignments that the participants work with in their everyday setting. Written feedback was provided on all assignments. The participants could also ask questions through a secure message system. | Sample: 13 patients  Sampling: Not stated  Data: Interviews  Analysis: Thematic analysis  Quality score: + | Patient +ve experiences:  *“Psychosocial support”*  *“Pt-staff communication”*  *“Feels looked after”*  *“Follow up / co-ordination of care”*  *“Clinical knowledge”*  *“Confidence/ motivation in self-care”*  *“Involvement in self-care”*  *“Improvement from usual care”*  *“User friendly”*  *“New technology”*  *“Saves travel time”*  *“Flexible”*  *“Comfort / Freedom at home”*  Patient –ve experiences:  *“Lack of pt-staff communication”*  *“Responsibility = anxiety”*  *“Lack of feedback”*  *“No effect on self-care”*  *“Lack of improvement”*  *“Technical difficulties”*  *“Medical jargon”*  *“Not user friendly”*  *“Technology overwhelming”*  *“Lack of education”*  *“Irrelevant training”*  *“Lack of privacy / security”*  *“Lack of support for care”*  *“Extra work = tiring”* |
| Lyngå 2013 [29]: Perceptions of transmission of body weight and telemonitoring in patients with heart failure?  Aim: The aim of this study was to explore and describe patients perceptions of transmission of body weight (BW) and telemonitoring, regularly accomplished from patients homes to an HF clinic  Country: Sweden  Funding: Not funded | Name: Wireless electronic scale  Type: Remote monitoring (Telemonitoring) and self-monitoring  Setting: Patient’s home  Duration: Not stated  Details: Patients in the intervention group were given an electronic scale to place in their home and asked to weigh themselves daily. The weight measurements were automatically trans- mitted to a heart failure clinic. If a weight gain was detected of 2 kg in 3 days, the patients were contacted by telephone. If there were signs of deterioration in the patients (e.g., increased breathlessness or tired- ness, swollen legs or difficulties to lay flat in bed, together with increased BW), the dose of diuretics was temporally increased. | Sample: 20 patients  Sampling: Purposive  Data: Interviews  Analysis: Phenomenology  Quality score: ++ | Patient +ve experiences:  *“Patient-staff communication”*  *“Feels looked after”*  *“Clinical knowledge”*  *“Confidence/ motivation in self-care”*  *“Involvement in self-care”*  *“Confidence in management decisions”*  *“User friendly”*  *“New technology”*  Patient –ve experiences:  *“Lack of patient-staff communication”*  *“Responsibility = anxiety”*  *“Lack of feedback”*  *“No effect on self-care”*  *“Technical difficulties”* |
| Nanevicz 2000 [30]: The feasibility of a telecommunications service in support of outpatient congestive heart failure care in a diverse patient population  Aim: We report on the feasibility and efficacy of using a home telemonitoring service as an adjunct to the outpatient care of heart failure patients from diverse backgrounds  Country: USA  Funding: American Heart Association | Name: Telemonitoring service provided by Alere Medical Inc., San Francis- co, CA  Type: Remote monitoring (Telemonitoring)  Setting: Patient’s home  Duration: 1-4 weeks  Details: The system combines a scale with a simple display monitor. The patient stood on the scale activating the monitor’s pre-recorded voice which told the patient his or her weight and how it compared to his or her ideal weight. The monitor then asked the patient a number of heart failure symptomatology questions (as defined by the physician), to which the patient responded by pressing ‘yes’ or ‘no’ on the monitor. Weight and symptom information was then transmitted over the telephone line to a nurse who contacted the patient if there was a weight gain of 5 lbs or a weight loss of 10 lbs within 5 days, or reported symptoms for 3 consecutive days. The nurse then notified the physician of any patient alerts or information. In addition, patients received weekly or biweekly calls from the nurse to review symptoms and medications, and to discuss an educational topic relevant to the patient’s medical history. The educational modules covered general information about heart failure, diet, medications, symptom recognition, exercise, and lifestyle. | Sample: 50 patients  Sampling: Purposive  Data: Questionnaires  Analysis: Not stated  Quality score: - | Patient +ve experiences:  *“Clinical knowledge”*  *“Popular”*  Patient –ve experiences:  *“Technical difficulties”*  Healthcare staff –ve experiences:  *“Increased workload”* |
| Näsström 2015 [62]: Heart failure patients' descriptions of participation in structured home care  Aim: To examine how heart failure patients receiving structured home care described participation in the care  Country: Sweden  Funding: Swedish Heart and Lung Association | Name: Structured home care – The Heart Failure at Home Model  Type: Home visits  Setting: Patient’s home  Duration: Not stated  Details: The patients received care that involved a multidisciplinary team with physicians and nurses, optimized treatment according to guidelines, educational strategies for patients/families/caregivers, and increased accessibility to care. The patients could contact the home care team at all hours | Sample: 19 patients  Sampling: Purposive  Data: Interviews  Analysis: Content analysis  Quality score: + | Patient +ve experiences:  *“Patient-patient communication”*  *“Patient-staff communication”*  *“Feels looked after”*  *“Clinical knowledge”*  *“Confidence/ motivation in self-care”*  *“Involvement in self-care”*  *“Confidence in management decisions”*  *“Improvement from usual care”*  Patient –ve experiences:  *“Reliance on staff”*  *“Lack of options”* |
| Odeh 2014 [31]: Implementing a telehealth service: nurses' perceptions and experiences  Aim: To elicit practice nurses' perceptions of the telehealth service  Country: UK  Funding: Not stated | Name: A remote patient monitoring (RPM) telehealth service  Type: Remote monitoring (Telemonitoring)  Setting: Workplace  Duration: Not stated  Details: The system consisted of a set of peripheral devices at the patient’s home, including blood-pressure monitor, pulse oximeter, thermometer, weighing scales and peak-flow monitor/spirometer. According to preapproved set plans, the patient takes the measurements and answers a series of questions about symptoms. The readings are transmitted to a monitoring centre where they are triaged. If the data fall outside the parameters set for the patient, a trigger will be generated and forwarded to the practice nurse. The nurse can also access the data remotely, enabling monitoring trends over time and making informed decisions accordingly. | Sample: 7 nurses  Sampling: Not stated  Data: Email interviews  Analysis: Thematic analysis  Quality score: + | Healthcare staff ­+ve experiences:  *“Familiarity/ communication with patient”*  *“Encourages patient self-care”*  Healthcare staff –ve experiences:  *“Lack of staff-staff communication”*  *“Not linked to records”*  *“Technical difficulties”*  *“Lack of equipment/ support”*  *“Lack of training”*  *“Increased workload”*  *“Slow to change practice”*  *“Lack of options”*  *“Patient selection decisions”*  *“Cost”*  *“Lack of evidence of effectiveness”* |
| Paget 2010 [32]: Using home telehealth to empower patients to monitor and manage long term conditions  Aim: Evaluation of the intervention  Country: Wales  Funding: Welsh assembly government grant | Name: Genesis home monitor  Type: Remote monitoring (Telemonitoring)  Setting: Patient’s home  Duration: 12 weeks  Details: The genesis monitor is a home device with a blood pressure cuff and pulse oximeter attached, and is also connected to a weighing scales. The monitor activates at a pre-set time daily and instructs the patient to take their measurements and answer yes/no questions about their symptoms set by a nurse. The patient can request a visit by the nurse from the monitor. The information is sent to a nurse via a telephone line, and the reports can be printed by the patient. | Sample: 22 patients, nurses (unknown number)  Sampling: Not stated  Data: Questionnaires  Analysis: Not stated  Quality score: - | Patient +ve experiences:  *“Feels looked after”*  *“Confidence/ motivation in self-care”*  *“Involvement in self-care”*  *“Comfort/ freedom at home”*  *“Clinical knowledge”*  *“User friendly”*  Patient –ve experiences:  *“Technical difficulties”*  *“Lack of support for care”*  Healthcare staff ­+ve experiences:  *“User friendly”*  Healthcare staff –ve experiences:  *“Technical difficulties”*  *“Increases patient concern/ confusion”*  *“Lack of equipment/ support”*  *“Increases errors”*  *“Lack of training”*  *“Slow to change practice”*  *“Increased workload*  *“Patient selection decisions”* |
| Payne 2015 [57]: Usability Testing of an Internet-Based e-Counselling Platform for Adults With Chronic Heart Failure  Aim: A prototype website evaluation to promote self-care in patients with heart failure via a counselling platform  Country: USA  Funding: Canadian Institutes of Health Research | Name: CHF-CePPORT - Canadian e-Platform to Promote Behavioural Self-Management in Chronic Heart Failure  Type: Online management  Setting: Clinic  Duration: 1 day  Details: 28 e-sessions delivered over a 12 month period consisting of a self-help video, educational content, interactive e-tools, and e-trackers for behaviour change. In this study, the participants were exposed to draft content to help refine it, not exposed to the completed 12-month package. | Sample: 7 patients  Sampling: Purposive  Data: Interviews and questionnaires  Analysis: Content analysis  Quality score: + | Patient +ve experiences:  *“Psychosocial support”*  *“Feels looked after”*  *“Clinical knowledge”*  Patient –ve experiences:  *“Not user friendly”* |
| Rahimpour 2008 [33]: Patients' perceptions of a home telecare system  Aim: To identify any major factors that could affect patients' perceptions of the intervention and use the findings to contribute to development of a theoretical framework for patient acceptance  Country: Australia  Funding: Funded by company developing intervention | Name: Home Telecare Management System (HTMS)  Type: Remote monitoring (Telemonitoring)  Setting: Hospital  Duration: 1 day  Details: The participants were shown a videotape demonstrating the range of clinical measurements the HTMS could provide: blood pressure, spirometry, temperature, weight, heart rate, ECG and providing feedback to patients including medication reminders and measurement scheduling. | Sample: 77 patients  Sampling: Purposive  Data: Focus groups  Analysis: Thematic analysis  Quality score: ++ | Patient +ve experiences:  *“Psychosocial support”*  *“Feels looked after”*  *“Clinical knowledge”*  *“Follow up/ co-ordination of care”*  *“Confidence/ motivation in self-care”*  *“Involvement in self-care”*  *“Confidence in management decisions”*  *“Improvement from usual care”*  *“User friendly”*  *“Popular”*  *“Saves travel time”*  *“Comfort/ freedom at home”*  Patient –ve experiences:  *“Lack of patient-staff communication”*  *“Responsibility = anxiety”*  *“Technical difficulties”*  *“Not user friendly”*  *“Technology overwhelming”*  *“Lack of training”*  *“Cost”*  *“Lack of options”*  *“Extra work for clinicians”* |
| Riley 2013 [34]: Does telemonitoring in heart failure empower patients for self-care? A qualitative study  Aim: To explore the extent to which telemonitoring in patients with heart failure empowers them to self- care  Country: UK  Funding: National Institute for Health Research (NIHR) | Name: HOME-HF  Type: Remote monitoring (Telemonitoring)  Setting: Patient’s home  Duration: 3-6 months  Details: Patients in this study used a stand-alone telemonitoring system that required them to take measurements daily using a weighing scale, automated blood pressure cuff and pulse oximeter, and by responding to four symptom questions. This data was transmitted for review by a cardiac nurse in the hospital who responded to clinical change. | Sample: 15 patients  Sampling: Purposive  Data: Interviews  Analysis: Constant comparison  Quality score: ++ | Patient +ve experiences:  *“Patient-staff communication”*  *“Feels looked after”*  *“Clinical knowledge”*  *“Confidence/ motivation in self-care”*  *“Involvement in self-care”*  *“Confidence in management decisions”*  *“User friendly”*  *“New technology”*  *“Flexible”*  *“Comfort/ freedom at home”*  Patient –ve experiences:  *“Reliance on staff”*  *“Lack of patient-staff communication”*  *“No effect on self-care”*  *“Lack of improvement”*  *“Not user friendly”* |
| Sanders 2012 [35]: Exploring barriers to participation and adoption of telehealth and telecare within the Whole System Demonstrator trial: a qualitative study  Aim: Examining withdrawal or non-involvement in trials, examining reasons people don't participate in some trials.  Country: UK  Funding: Department of Health | Name: Not stated  Type: Remote monitoring (Telemonitoring)  Setting: Patient’s home  Duration: 12 months  Details: Telehealth equipment included a monitor unit via which recordings from peripheral devices (measuring blood pressure, blood glucose, blood oxygen level, weight, and peak flow) were to be uploaded to a monitoring centre. Telecare interventions also included various sensors to detect gas, water overflow, falls and movement around the property. Such sensors would trigger alarms direct to a monitoring centre if anything abnormal was detected, allowing emergency intervention. | Sample: 21 patients  Sampling: Convenience  Data: Observation and interviews  Analysis: Constant comparison  Quality score: ++ | Patient –ve experiences:  *“Reliance on staff”*  *“Lack of patient-staff communication”*  *“Unpredictable clinical management”*  *“Lack of improvement”*  *“Technical difficulties”*  *“Not user friendly”*  *“Technology overwhelming”*  *“Lack of training”*  *“Extra work = tiring”*  *“Lack of efficiency/ co-ordination”*  *“Cannot replace hospital care”*  *“Threat to independence/ control”*  *“Technology not needed”*  Healthcare staff +ve experiences:  *“Change is stressful”*  *“Technology not the solution”* |
| Selman 2015 [60]: Appropriateness and acceptability of a Tele-Yoga intervention for people with heart failure and chronic obstructive pulmonary disease: qualitative findings from a controlled pilot study  Aim: To assess acceptability and appropriateness of the intervention, educational control and study design.  Country: USA  Funding: University of California, San Francisco, NIHR Biomedical Research Centre for Mental Health, Guys and St Thomas NHS Foundation Trust, and Kings College London | Name: Tele-Yoga  Type: Educational tool  Setting: Patient’s home  Duration: 1-3 months  Details: Home-based one-hour Tele-Yoga classes were offered twice weekly for 8 weeks. Classes integrated held postures, breathing exercises, imagery, meditation and relaxation. The teacher modified postures as needed to meet the physical ability of each participant and offered corrections and adjustments with verbal cues, further explanation, and modelling of poses to participants. Participants were provided with a yoga mat if needed, and encouraged to use common household items as props where necessary. | Sample: 15 patients  Sampling: Convenience  Data: Interviews and questionnaires  Analysis: Thematic analysis  Quality score: + | Patient +ve experiences:  *“Confidence/ motivation in self-care”*  *“Involvement in self-care”*  *“User friendly”*  *“Comfort/ freedom at home”*  Patient –ve experiences:  *“No effect on self-care”*  *“Technical difficulties”*  *“Not user friendly”* |
| Seto 2010 [43]: Attitudes of Heart Failure Patients and Health care Providers towards Mobile Phone-Based Remote Monitoring  Aim: To assess the attitudes of heart failure patients and their health care providers from a heart function clinic in a large urban teaching hospital toward the use of mobile phone-based remote monitoring  Country: Canada  Funding: Toronto General Hospital Foundation and the NSERC Strategic Research Network Grant entitles Healthcare Support through Information Technology Enhancements (hSITE) | Name: Mobile-phone based remote monitoring  Type: Remote monitoring (Telemonitoring) and self-monitoring  Setting: Patient’s home  Duration: Not stated  Details: Patient and clinician views (via survey and interview) of a description of a mobile phone-based remote monitoring system including a prototype system demonstrating the steps they would have to take for the proposed monitoring system. This included a wireless scale, blood pressure monitor and electrocardiogram. Patients would be required to monitor their own weight, blood pressure, and symptoms daily. Depending upon readings an alert message could be generated and sent to the patient and the clinician via a secure password-protected website. | Sample: 114 patients, 16 clinicians  Sampling: Consecutive  Data: Interviews and questionnaires  Analysis: Content analysis  Quality score: ++ | Patient +ve experiences:  *“Feels looked after”*  *“Confidence/ motivation in self-care”*  *“Involvement in self-care”*  *“Saves travel time”*  *“Flexible”*  Patient –ve experiences:  *“Not user friendly”*  *“Technology overwhelming”*  *“Unreliable information”*  *“Lack of privacy/ security”*  *“Extra work for clinicians”*  Healthcare staff +ve experiences:  *“Pro-active management”*  *“Encourages patient self-care”*  *“Save travel time”*  Healthcare staff –ve experiences:  *“Not user friendly”*  *“Increased workload”*  *“Increases errors”*  *“Medicolegal concerns”*  *“Information privacy/ security”* |
| Seto 2012 [42]: Perceptions and experiences of heart failure patients and clinicians on the use of mobile phone-based telemonitoring  Aim: To determine features that enable successful telemonitoring  Country: Canada  Funding: Toronto general hospital foundation and the natural sciences and engineering research council of Canada | Name: Custom built software on mobile phone  Type: Remote monitoring (Telemonitoring)  Setting: Patient’s home  Duration: 6 months  Details: Patients were given equipment to measure weight and blood pressure daily and answer symptom questions on a mobile phone. They were also asked to record their ECG weekly using a recorder provided. The measurements were sent from the devices to a blackberry mobile device via Bluetooth, which was transmitted to the hospital server. Instructions or alerts can be sent to the patient, and all information was available on a website. Alerts with new information was emailed to the physician’s mobile phone if measurements were outside a target range or symptoms reported. | Sample: 22 patients, 5 clinicians  Sampling: Purposive  Data: Interviews  Analysis: Content analysis  Quality score: ++ | Patient +ve experiences:  *“Involvement in self-care”*  *“Clinical knowledge”*  *“Feels looked after”*  *“Patient – staff communication”*  *“Increased confidence and motivation in self-care”*  *“User friendly”*  *“Saves travel time”*  *“Flexible”*  *“Improvement from usual care”*  Patient –ve experiences:  *“Language barrier”*  *“Cost”*  *“Lack of privacy/ security”*  *“Responsibility = anxiety”*  *“Reliance on staff”*  Carer +ve experiences:  *“Feels patient is looked after”*  Healthcare staff –ve experiences:  *“Improvement from usual care”*  *“Confidence in management”*  *“Familiarity/ communication with patient”*  *“Proactive management”*  *“Encourages patient self-care”*  *“Increases patient knowledge”*  *“Saves travel time”*  *“Better knowledge of patient status”*  *“Cost savings”*  *“Flexible to practice”*  Healthcare staff –ve experiences:  *“Cost”*  *“Increased workload”*  *“Not linked to records”*  *“Lack of equipment/ support”* |
| Seto 2019 [49]: Implementation of a heart failure telemonitoring system in home care nursing: Feasibility study  Aim: To determine the feasibility of implementing a mobile phone-based TM system through a home care nursing agency and to explore the feasibility of conducting a future effectiveness trial.  Country: Canada  Funding: ParaMed Home Health Care, CellTrak Technologies Inc, Natural Sciences and Engineering Research Council of Canada Strategic Research Network | Name: Mobile phone based TM system  Type: Remote monitoring (Telemonitoring)  Setting: Patient’s home  Duration: 3-6 months  Details: Through a mobile phone app, the TM system allows HF patients to monitor their health by recording weight and blood pressure measurements daily with Bluetooth-enabled home medical devices. Patients are asked to answer simple yes or no symptom questions on the mobile phone, such as whether they have more chest pain than usual or if they have more difficulty breathing at night than usual. Automated self-care instructions and advice are sent immediately to the patient based on their measurements and reported symptoms. If the TM system detected signs of an exacerbation, an alert with all relevant data was sent to the appropriate nurse’s mobile phone through the software that is part of the integrated care coordination platform. The nurse and patient’s physician were able to access all the patient’s TM data through a clinical dashboard through a secure website. The alert threshold values can also be set and modified through the clinical dashboard. | Sample: 6 patients, over 50 other staff including various home care nurses, portal administrators, assignment coordinators, nursing supervisors, off-hours supervisors  Sampling: Convenience  Data: Interviews, self-completion questionnaire, secondary data  Analysis: Thematic and framework analysis  Quality score: ++ | Patient +ve experiences:  *“Pt-staff communication”*  *“Clinical knowledge”*  *“Follow up / co-ordination of care”*  *“Confidence and motivation in self-care”*  *“User friendly”*  Patient –ve experiences:  *“Responsibility = anxiety”*  *“Unpredictable clinical management”*  *“Technical difficulties*  *“Technology overwhelming”*  *“Unreliable information”*  *“Intrusive”*  *“Extra work = tiring”*  *“Uncomfortable at home”*  *“Extra work for clinicians”*  Healthcare staff +ve experiences:  *“Familiarity/ communication with patient”*  *“Better knowledge of patient status”*  *“Proactive management”*  *“Identifies priorities in care”*  *“Improvement from usual care”*  *“Encourages patient self-care”*  *“Increases patient knowledge”*  *“User friendly”*  *“Automated saves time”*  *“Saves travel time”*  Healthcare staff –ve experiences:  *“Increases patient concern / confusion”*  *“Lack of pt-staff communication”*  *“Lack of staff-staff communication”*  *“Inflexible guidance”*  *“Not user friendly”*  *“Technical difficulties”*  *“Lack of equipment / support”*  *“Cost”*  *“Increased workload”*  *“Lack of options*  *“Patient selection decisions”*  *“Limits clinical care”*  *“Lack of confidence in equipment / readings”*  *“Clinician anxiety”* |
| Seuren 2020 [50]: Physical Examinations via Video for Patients With Heart Failure: Qualitative Study Using Conversation Analysis  Aim: To explore the opportunities and challenges of remote physical examination of patients with heart failure using video-mediated communication technology.  Country: UK  Funding: National Institute for Health Research, Wellcome Trust | Name: FaceTime (Apple Inc)  Type: Remote monitoring (Telemonitoring)  Setting: Patient’s home  Duration: Not stated  Details: Video consultations (using FaceTime) between heart failure specialist nurses and community-based patients having routine heart failure reviews, including physical examinations (typically measuring weight, blood pressure, heart rate, and rhythm [using a blood pressure monitor put on by the patient or relative and incorporating irregular heartbeat indicator to assess for atrial fibrillation] and oxygen saturation; assessing oedema in ankles and legs; and performing chest auscultation for signs of fluid overload or infection). Jugular venous pressure is not generally assessed by heart failure specialist nurses. The alert threshold values can also be set and modified through the clinical dashboard. | Sample: 5 nurses, 7 patients  Sampling: Not stated  Data: Interviews  Analysis: Content analysis  Quality score: + | Patient +ve experiences:  *“Confidence and motivation in self-care”*  *“Involvement in self-care”*  Patient –ve experiences:  *“Reliance on staff”*  *“Technical difficulties”*  *“Not user friendly”*  *“Technology overwhelming”*  *“Lack of training”*  *“Threat to independence / control”*  Carer –ve experiences  *“Causes concern for patient”*  Healthcare staff –ve experiences:  *“Not user friendly”*  *“Computer literacy”*  *“Increases errors”*  *“Limits clinical care”* |
| Sharma 2010 [44]: Clinical users' perspective on telemonitoring of patients with long term conditions: understood through concepts of Giddens's structuration theory & consequence of modernity  Aim: To understand initial thoughts of clinical users about the newly introduced telehealth service  Country: UK  Funding: MATCH (Multidisciplinary Assessment of Technologies Centre for Healthcare) | Name: Not stated  Type: Remote monitoring (Telemonitoring)  Setting: Patient’s home  Duration: Not stated  Details: The clinical users participating in this study were involved in delivering care to patients who were taking part in a randomised controlled trial, evaluating clinical effectiveness of telemonitoring of patients with chronic conditions such as heart failure and obstructive pulmonary disease. | Sample: 16 healthcare staff  Sampling: Not stated  Data: Focus groups  Analysis: Thematic analysis  Quality score: - | Healthcare staff –ve experiences:  *“Increases patient concern/ confusion”*  *“Lack of equipment/ support”*  *“Lack of training”*  *“Increases workload”*  *“Slow to change practice”*  *“Increases errors”*  *“Patient selection decisions”*  *“Limits clinical care”*  *“Lack of confidence in equipment/ readings”*  *“Clinician anxiety”* |
| Sharma 2014 [45]: Nurses' and community support workers' experience of telehealth: a longitudinal case study  Aim: Introduction of telehealth into the healthcare setting has been recognised as a service that might be experienced as disruptive. This paper explores how this disruption is experienced  Country: UK  Funding: Not stated | Name: Not stated  Type: Remote monitoring (Telemonitoring)  Setting: Workplace  Duration: Not stated  Details: Not stated | Sample: 20 clinicians  Sampling: Not stated  Data: Focus groups and interviews  Analysis: Interpretive phenomenological analysis  Quality score: + | Healthcare staff +ve experiences:  *“Familiarity/ communication with patient”*  *“Encourages teamwork”*  *“Identifies priorities in care”*  *“Improvement from usual care”*  *“Technical support”*  Healthcare staff –ve experiences:  *“Increases patient concern/ confusion”*  *“Lack of patient-staff communication”*  *“Lack of staff-staff communication”*  *“Not linked to records”*  *“Lack of equipment/ support”*  *“Lack of training”*  *“Increased workload”*  *“Slow to change practice”*  *“Increases errors”*  *“Health inequality”*  *“Limits clinical care”*  *“Lack of confidence in equipment/ readings”* |
| Stromberg 2002 [61]: Interactive education on CD-ROM-a new tool in the education of heart failure patients  Aim: To develop and evaluate whether a computer-based program for patients with heart failure was user-friendly, could be operated by elderly patients and give sufficient information about heart failure  Country: Sweden  Funding: Aventis Pharma | Name: Not stated  Type: Educational tool  Setting: Hospital  Duration: 1 day  Details: The final programme constituted eight modules, seven of which were educational relating to heart failure and one which was a self-test. After completing the education the patients could perform a self-test which gave a green or red light and results were shown graphically. | Sample: 42 patients, 3 nurses  Sampling: Not stated  Data: Questionnaire  Analysis: Not specified  Quality score: - | Patient +ve experiences:  *“Clinical knowledge”*  *“Flexible”*  Patient –ve experiences:  *“Technology overwhelming”*  *“Lack of education”*  Healthcare staff +ve experiences:  *“Familiarity/ communication with patient”*  *“Increases patient knowledge”*  *“Automated saves time”*  Healthcare staff –ve experiences:  *“Lack of patient-staff communication”*  *“Not user friendly”* |
| Svagård 2014 [36]: A usability study of a mobile monitoring system for congestive heart failure patients  Aim: Usability study of intervention  Country: USA  Funding: Telemedicine and Advanced Technology Research Centre | Name: The ESUMS system  Type: Remote monitoring (Telemonitoring)  Setting: Patient’s home  Duration: 3 weeks  Details: Two devices: 1) wearable sensor belt that measures heart rate, activity level, posture and skin temperature 2) a smartphone for viewing and transmitting data to a central server. If the user reaches pre-set activity goals for the day, a “motivation heart” appears on the phone. Also connects with other sensor devices (e.g. pulse oximeter). A pc-application retrieves the data from the server and is used by the supervising nurse or doctor for follow-up from a remote site. | Sample: 5 heart failure patients, 4 nurses  Sampling: Not stated  Data: Interviews  Analysis: Not stated  Quality score: - | Patient +ve experiences:  *“Confidence/ motivation in self-care”*  *“Popular”*  *“User friendly”*  Patient –ve experiences:  *“Not user friendly”*  *“Extra work = tiring”*  *“Technical difficulties”*  *“Responsibility = anxiety”*  Healthcare +ve experiences:  *“Increases patient knowledge”*  *“Encourages patient self-care”*  *“User friendly”*  *“Better knowledge of patient status”*  Healthcare –ve experiences:  *“Technical difficulties”*  *“Lack of options”* |
| Taylor 2015 [37]: Examining the use of telehealth in community nursing: identifying the factors affecting frontline staff acceptance and telehealth adoption  Aim: To examine frontline staff acceptance of telehealth and identify barriers to and enablers of successful adoption of remote monitoring for patients with Chronic Obstructive Pulmonary Disease and Chronic Heart Failure  Country: UK  Funding: Assisted Living Innovation Platform | Name: Not stated  Type: Remote monitoring (Telemonitoring)  Setting: Patient’s home  Duration: Not stated  Details: Case studies of four community health services in England that use telehealth to monitor patients with Chronic Obstructive Pulmonary Disease and Chronic Heart Failure. | Sample: 105 healthcare staff  Sampling: Purposive  Data: Interviews  Analysis: Thematic analysis  Quality score: ++ | Healthcare +ve experiences:  *“Encourages teamwork”*  *“Staff education”*  *“Automated saves time”*  *“Local champions”*  Healthcare –ve experiences:  *“Technical difficulties”*  *“Lack of equipment/ support”*  *“Lack of training”*  *“Increased workload”*  *“Slow to change practice”*  *“Patient selection decisions”*  *“Cost”*  *“Lack of confidence in equipment/ readings”*  *“Clinician anxiety”*  *“Lack of evidence of effectiveness”* |
| Whitten 1998 [39]: Nurse reactions to a prototype home telemedicine system  Aim: To study nurse reactions to a home telemedicine system  Country: USA  Funding: National telecommunications information administration | Name: HELP Innovations telemedicine system  Type: Remote monitoring (Telemonitoring)  Setting: Patient’s home  Duration: Not stated  Details: Not stated | Sample: 4 nurses  Sampling: convenience  Data: Interviews  Analysis: Content analysis  Quality score: - | Healthcare +ve experiences:  *“Increases patient knowledge”*  *“Encourages patient self-care”*  *“ Familiarity/ communication with patient”*  Healthcare –ve experiences:  *“Computer Literacy”*  *“Not user friendly”*  *“Technical difficulties”*  *“Lack of equipment/support”*  *“Lack of patient-staff communication”*  *“Limits clinical care”* |
| Whitten 2009 [38]: St. Vincent's Home telehealth for congestive heart failure patients  Aim: To study whether telehome health patients exhibit enhanced clinical outcomes and patient perceptions of telehome healthcare  Country: USA  Funding: Regenstrief Foundation | Name: St. Vincent Telehome Project  Type: Remote monitoring (Telemonitoring)  Setting: Patient’s home  Duration: 1-3 months  Details: The telehome project consisted of telehealth devices for patients to record their own weight and blood pressure using electronic scales and blood pressure monitor. Data was transferred via telephone for review by a nurse. | Sample: 35 patients  Sampling: Convenience  Data: Interviews  Analysis: Not stated  Quality score: - | Patient +ve experiences:  *“Feels looked after”*  *“Clinical knowledge”*  *“Confidence/ motivation in self-care”*  *“User friendly”*  *“Popular”*  *“Comfort/ freedom at home”*  Patient –ve experiences:  *“Technical difficulties”*  *“Cannot replace hospital care”* |
| Woodend 2008 [40]: Telehome monitoring in patients with cardiac disease who are at high risk of readmission  Aim: To determine whether telehome monitoring of patients with cardiac disease at high risk of readmission would reduce hospital readmissions, improve functional status, and improve quality of life over usual care  Country: USA  Funding: Merck-Frosst Canada | Name: Telehome  Type: Remote monitoring (Telemonitoring)  Setting: Patient’s home  Duration: 1-3 months  Details: 3 months of video conferencing with a nurse, daily weight and blood pressure, and periodic ECG. Video conferences included an assessment of the patient’s progress and self-care education by the telehome-care nurse. The educational content was covered within the first 8 weeks of monitoring. All patients were given a 24-7 telephone number to access an advanced practice nurse with questions related to their care. | Sample: 249 patients  Sampling: Convenience  Data: Questionnaires  Analysis: Not specified  Quality score: - | Patient +ve experiences:  *“Feels looked after”*  *“Clinical knowledge”*  *“Confidence/ motivation in self-care”*  Patient –ve experiences:  *“Technical difficulties”*  *“Technology overwhelming”*  *“Cost”*  *“Threat to independence/ control”* |
| Young 2008 [63]: A "basket of care" for heart failure patients managing at home: evaluating a community-based nursing intervention from a patient's perspective  Aim: The researchers aimed to answer the question; What is the patients’ perception of care received in the CH-CSSS-CSHFP?  Country: Canada  Funding: Not funded | Name: The Centre Hospitalier—  Centre de Santé et de Services Sociaux—Corridor of Service for Heart Failure Patients (CH-CSSS-CSHFP)  Type: Community care  Setting: Patient’s home  Duration: Not stated  Details: Community-based nursing intervention is based on six pillars of HF self-care including education, medication, nutrition, signs and symptoms, psychosocial issues, and coordination of health care services. | Sample: 5 patients  Sampling: Purposive  Data: Interviews  Analysis: Grounded theory  Quality score: + | Patient +ve experiences:  *“Patient-staff communication”*  *“Feels looked after”*  *“Follow up/ co-ordination of care”*  *“Confidence in management decisions”*  *“Improvement from usual care”*  *“Comfort/ freedom at home”*  Patient –ve experiences:  *“Lack of patient-staff communication”*  *“Lack of improvement”* |
| Zulman 2015 [67]: How Can eHealth Technology Address Challenges Related to Multimorbidity? Perspectives from Patients with Multiple Chronic Conditions  Aim: 1) Identify self-care and health access issues of patients multimorbidities; 2) Identify opportunities to support these patients through eHealth  Country: USA  Funding: the Gordon & Betty Moore Foundation | Name: Not stated  Type: Not stated  Setting: Clinic  Duration: Not stated  Details: Focus groups for the development of a remote care intervention for patients with multiple chronic conditions. | Sample: 53 patients  Sampling: Purposive  Data: Focus groups  Analysis: Content analysis  Quality score: ++ | Patient +ve experiences:  *“Patient-staff communication”*  *“Feels looked after”*  *“Follow up/ co-ordination of care”*  *“Confidence in management decisions”*  *“Improvement from usual care”*  *“Comfort/ freedom at home”*  Patient –ve experiences:  *“Lack to patient-staff communication”*  *“Lack of improvement”* |
