# Supplementary material for: Factors Affecting Patient and Physician Engagement in Remote Health Care for Heart Failure: Systematic Review
Source: JMIR Cardio. 2022 Apr 6;6(1):e33366. doi: 10.2196/33366 (PMC9021943; doi:10.2196/33366)
Supplement: Multimedia Appendix 4 [file cardio_v6i1e33366_app4.doc]

**Multimedia Appendix 4**. NICE quality appraisal

|  | *Theoretical approach* | | *Methodology* | | *Trustworthiness* | | | *Analysis* | | | | | | *Ethics* | Overall rating |
| --- | --- | --- | --- | --- | --- | --- | --- | --- | --- | --- | --- | --- | --- | --- | --- |
|  | Qualitative approach | Study purpose | Study design | Data collection | Role of researcher | Context | Reliable methods | Rigorous data analysis | Rich data | Reliable analysis | Convincing findings | Relevant findings | Conclusions | Clear and coherent reporting |
| Abidi 2013 [51] | Appropriate | Clear | Defensible | Appropriate | NR | Clear | Not sure | Rigorous | Poor | Reliable | Not convincing | Irrelevant | Adequate | Appropriate | + |
| Agrell 2000 [18] | Appropriate | Clear | Defensible | Appropriate | NR | Unclear | Unreliable | Not rigorous | Poor | Unreliable | Not convincing | Relevant | Adequate | Inappropriate | + |
| Akyar 2019 [58] | Appropriate | Clear | Defensible | Appropriate | Unclear | Clear | Not sure | Rigorous | Rich | Not sure | Convincing | Relevant | Adequate | Not sure | ++ |
| Barron 2013 [54] | Appropriate | Clear | Indefensible | Inappropriate | NR | Clear | Reliable | Not rigorous | Poor | Unreliable | Not convincing | Relevant | Inadequate | Inappropriate | - |
| Bartlett 2014 [19] | Appropriate | Clear | Defensible | Appropriate | NR | Not sure | Unreliable | Rigorous | Rich | Reliable | Convincing | Relevant | Adequate | Appropriate | + |
| Bekelman 2014 [64] | Appropriate | Clear | Defensible | Appropriate | Unclear | Unclear | Not sure | Rigorous | Poor | Not sure | Not sure | Relevant | Adequate | Inappropriate | + |
| Cajita 2018 [46] | Appropriate | Clear | Defensible | Appropriate | Unclear | Clear | Reliable | Rigorous | Rich | Reliable | Convincing | Relevant | Adequate | Appropriate | ++ |
| Chantler 2016 [68] | Appropriate | Clear | Defensible | Appropriate | NR | Clear | Reliable | Rigorous | Rich | Reliable | Convincing | Relevant | Adequate | Appropriate | ++ |
| Dang 2017 [69] | Appropriate | Clear | Not sure | Not sure | NR | Unclear | Not sure | Not rigorous | Poor | Unreliable | Not sure | Partially relevant | Not sure | Appropriate | - |
| Dinesen 2008 [20] | Appropriate | Clear | Defensible | Appropriate | Unclear | Clear | Reliable | Rigorous | Poor | Reliable | Convincing | Relevant | Adequate | Appropriate | ++ |
| Dubois 2001 [21] | Appropriate | Clear | Defensible | Appropriate | Clear | Clear | Reliable | Not rigorous | Poor | Unreliable | Convincing | Relevant | Adequate | Appropriate | + |
| Earnest 2004 [55] | Appropriate | Clear | Defensible | Appropriate | NR | Not sure | Reliable | Rigorous | Poor | Reliable | Convincing | Relevant | Adequate | Appropriate | ++ |
| Fairbrother 2014 [22] | Appropriate | Clear | Defensible | Appropriate | Clear | Clear | Reliable | Rigorous | Rich | Reliable | Convincing | Relevant | Adequate | Appropriate | ++ |
| Finkelstein 2011 [23] | Appropriate | Clear | Defensible | Not sure | NR | Unclear | Not sure | Not rigorous | Poor | Unreliable | Not sure | Irrelevant | Inadequate | Inappropriate | - |
| Grace 2017 [47] | Appropriate | Clear | Defensible | Appropriate | NR | Clear | Reliable | Rigorous | Rich | Reliable | Convincing | Relevant | Adequate | Not sure | ++ |
| Green 2006 [52] | Appropriate | Clear | Defensible | Appropriate | NR | Not sure | Not sure | Rigorous | Rich | Not sure | Convincing | Relevant | Adequate | Inappropriate | + |
| Gund 2008 [24] | Appropriate | Clear | Indefensible | Appropriate | NR | Unclear | Unreliable | Not rigorous | Poor | Reliable | Convincing | Relevant | Adequate | Inappropriate | - |
| Hall 2014 [41] | Appropriate | Clear | Defensible | Appropriate | NR | Not sure | Reliable | Rigorous | Rich | Reliable | Convincing | Relevant | Adequate | Not sure | + |
| Heckemann 2016 [48] | Appropriate | Clear | Defensible | Appropriate | Clear | Clear | Reliable | Rigorous | Rich | Not sure | Convincing | Relevant | Adequate | Appropriate | ++ |
| Heisler 2007 [65] | Not sure | Clear | Defensible | Appropriate | Unclear | Unclear | Not sure | Not rigorous | Poor | Reliable | Convincing | Relevant | Not sure | Not sure | + |
| Hunting 2015 [25] | Appropriate | Clear | Defensible | Appropriate | Clear | Clear | Reliable | Rigorous | Rich | Reliable | Convincing | Relevant | Adequate | Appropriate | ++ |
| Johnston 2010 [26] | Appropriate | Unclear | Defensible | Not sure | NR | Unclear | Unreliable | Not rigorous | Poor | Unreliable | Not sure | Irrelevant | Inadequate | Inappropriate | - |
| Kenealy 2015 [27] | Appropriate | Clear | Defensible | Appropriate | NR | Clear | Not sure | Rigorous | Rich | Reliable | Convincing | Relevant | Adequate | Appropriate | ++ |
| Leslie 2006 [53] | Appropriate | Clear | Not sure | Not sure | NR | Not sure | Not sure | Not rigorous | Poor | Not sure | Not convincing | Relevant | Not sure | Not sure | - |
| Lind 2014 [28] | Appropriate | Clear | Defensible | Appropriate | NR | Unclear | Unreliable | Not rigorous | Poor | Unreliable | Convincing | Relevant | Not sure | Appropriate | + |
| Lowrie 2014 [66] | Appropriate | Clear | Defensible | Appropriate | NR | Not sure | Not sure | Not rigorous | Rich | Not sure | Convincing | Partially relevant | Adequate | Not sure | + |
| Lundgren 2015 [56] | Appropriate | Clear | Indefensible | Appropriate | NR | Not sure | Not sure | Not rigorous | Poor | Reliable | Not convincing | Relevant | Not sure | Appropriate | + |
| Lundgren 2018 [59] | Appropriate | Clear | Defensible | Appropriate | Clear | Clear | Reliable | Rigorous | Rich | Reliable | Convincing | Relevant | Adequate | Appropriate | ++ |
| Lynga 2013 [29] | Appropriate | Clear | Defensible | Appropriate | NR | Not sure | Reliable | Rigorous | Rich | Reliable | Convincing | Relevant | Adequate | Appropriate | ++ |
| Nanevicz 2000 [30] | Not sure | Clear | Defensible | Appropriate | NR | Clear | Unreliable | Rigorous | Not sure | Not sure | Not sure | Relevant | Adequate | Appropriate | - |
| Nasstrom 2015 [62] | Appropriate | Clear | Defensible | Appropriate | Unclear | Clear | Not sure | Rigorous | Rich | Reliable | Convincing | Relevant | Adequate | Appropriate | + |
| Odeh 2014 [31] | Appropriate | Clear | Not sure | Not sure | NR | Unclear | Not sure | Rigorous | Rich | Unreliable | Convincing | Relevant | Adequate | Not sure | + |
| Paget 2010 [32] | Appropriate | Clear | Indefensible | Inappropriate | NR | Unclear | Unreliable | Not rigorous | Poor | Unreliable | Not convincing | Relevant | Not sure | Inappropriate | - |
| Payne 2015 [57] | Appropriate | Clear | Defensible | Appropriate | Clear | Clear | Not sure | Not rigorous | Not sure | Reliable | Not sure | Relevant | Adequate | Appropriate | + |
| Rahimpour 2008 [33] | Appropriate | Clear | Defensible | Appropriate | Clear | Not sure | Reliable | Rigorous | Not sure | Reliable | Convincing | Relevant | Adequate | Appropriate | ++ |
| Riley 2013 [34] | Appropriate | Clear | Defensible | Appropriate | NR | Clear | Reliable | Rigorous | Rich | Reliable | Convincing | Relevant | Adequate | Appropriate | ++ |
| Sanders 2012 [35] | Appropriate | Clear | Defensible | Appropriate | Unclear | Clear | Reliable | Rigorous | Not sure | Reliable | Convincing | Relevant | Adequate | Appropriate | ++ |
| Selman 2015 [60] | Appropriate | Clear | Defensible | Appropriate | Unclear | Not sure | Unreliable | Not sure | Not sure | Reliable | Convincing | Relevant | Adequate | Appropriate | + |
| Seto 2010 [43] | Appropriate | Clear | Defensible | Appropriate | Unclear | Clear | Reliable | Rigorous | Rich | Reliable | Convincing | Relevant | Adequate | Appropriate | ++ |
| Seto 2012 [42] | Appropriate | Clear | Defensible | Appropriate | NR | Clear | Reliable | Rigorous | Rich | Reliable | Convincing | Relevant | Adequate | Inappropriate | ++ |
| Seto 2019 [49] | Appropriate | Clear | Defensible | Appropriate | Unclear | Clear | Not sure | Rigorous | Rich | Not sure | Convincing | Relevant | Adequate | Appropriate | ++ |
| Seuren 2020 [50] | Appropriate | Clear | Not sure | Not sure | NR | Clear | Not sure | Not sure | Rich | Not sure | Convincing | Relevant | Adequate | Appropriate | + |
| Sharma 2010 [44] | Appropriate | Clear | Not sure | Not sure | NR | Clear | Not sure | Rigorous | Rich | Not sure | Convincing | Relevant | Adequate | Not sure | - |
| Sharma 2014 [45] | Appropriate | Clear | Not sure | Appropriate | Unclear | Not sure | Not sure | Rigorous | Not sure | Not sure | Convincing | Relevant | Adequate | Appropriate | + |
| Stromberg 2002 [61] | Appropriate | Clear | Not sure | Not sure | NR | Clear | Not sure | Not rigorous | Not sure | Not sure | Not sure | Relevant | Adequate | Not sure | - |
| Svagard 2014 [36] | Appropriate | Clear | Not sure | Not sure | Unclear | Unclear | Not sure | Not sure | Poor | Not sure | Not sure | Relevant | Adequate | Inappropriate | - |
| Taylor 2015 [37] | Appropriate | Clear | Defensible | Appropriate | NR | Clear | Reliable | Rigorous | Rich | Reliable | Convincing | Relevant | Adequate | Appropriate | ++ |
| Whitten 1998 [39] | Appropriate | Clear | Not sure | Not sure | Unclear | Unclear | Not sure | Not sure | Poor | Not sure | Convincing | Relevant | Adequate | Inappropriate | - |
| Whitten 2009 [38] | Not sure | Clear | Indefensible | Not sure | NR | Unclear | Unreliable | Not sure | Poor | Not sure | Not sure | Relevant | Not sure | Not sure | - |
| Woodend 2008 [40] | Not sure | Clear | Not sure | Appropriate | NR | Unclear | Unreliable | Not rigorous | Poor | Unreliable | Not sure | Partially relevant | Not sure | Appropriate | - |
| Young 2008 [63] | Appropriate | Clear | Not sure | Appropriate | Unclear | Clear | Reliable | Rigorous | Poor | Not sure | Convincing | Relevant | Adequate | Appropriate | + |
| Zulman 2015 [67] | Appropriate | Clear | Defensible | Appropriate | NR | Clear | Reliable | Rigorous | Rich | Reliable | Convincing | Relevant | Adequate | Appropriate | ++ |
