# Supplementary material for: Factors Affecting Patient and Physician Engagement in Remote Health Care for Heart Failure: Systematic Review
Source: JMIR Cardio. 2022 Apr 6;6(1):e33366. doi: 10.2196/33366 (PMC9021943; doi:10.2196/33366)
Supplement: Multimedia Appendix 5 [file cardio_v6i1e33366_app5.doc]

**Multimedia Appendix 5**: First order codes identified in studies included in systematic review, with associated descriptions.

| **PATIENT POSITIVE EXPERIENCES** | |
| --- | --- |
| Psychosocial support | The intervention encourages or promotes interaction with a support group such as friends, family or carers to provide social support for the patient and this can help with feelings of loneliness or isolation or mental health issues. |
| Patient-to-patient communication | Communication between patients in the same intervention allows peer support, advice and a feeling of togetherness within the group. |
| Patient-to-staff communication | Remote care strengthens connection between patient and staff and causes a desire for greater communication between two parties |
| “Feels looked after” | The feeling of being monitored or watched by health-care practitioners which makes patients feel reassured, at ease, and looked after, even when at home. |
| Clinical knowledge | The intervention provides increased knowledge and information about disease and health status, which is seen as helpful by patients. |
| Follow up/ co-ordination of care | The intervention allows continuity of care by means of sharing patient information across health disciplines and thus aids the co-ordination of care given to the patient. |
| Confidence/ motivation in self-care | Exposure to intervention builds patient confidence and motivation in making their own health-related decisions. |
| Involvement in self-care | Patients having an active role in intervention brings greater independence and sense of control over health. |
| Confidence in management decisions | Patients feel more confident in the assessment and management of clinicians while using the intervention as they feel it provides staff with more information to make better decisions. |
| Improvement from usual care | Patient feels the intervention is an improvement from usual care in terms of their recovery, symptoms and quality of life. |
| User friendly | Intervention is perceived as easy to use by patients. |
| New technology | Patients take an interest in new health technology and are keen to engage in a novel method of self-care. |
| Popular | Intervention is used by many patients. |
| Saves travel time | Remote monitoring saves patients from visits to the clinic, which is a benefit if they suffer from reduced mobility. |
| Flexible | The intervention has flexible elements which can fit around the patient's lifestyle and allows greater freedom with management of their disease. |
| Comfort/ Freedom at home | Patients feel more relaxed in their home environment and free to pursue other activities. |
| **PATIENT NEGATIVE EXPERIENCES** | |
| Reliance on staff | Patient sees staff as primary responsible person for their health management and the technology is a tool to help them. They do not experience increased independence as a result. |
| Lack of patient-to-patient communication | Lack of contact with other patients or support in the group leads to feelings of isolation. |
| Lack of patient-to-staff communication | Lack of options for patient dialogue with healthcare staff. |
| Responsibility = anxiety | Even with intervention, patients do not feel comfortable taking responsibility for their own health management, and having to do more for themselves causes anxiety. |
| Lack of feedback | Lack of feedback from the intervention via the equipment or the clinicians/staff guiding the intervention as to the patient's actions and performance with self-care. Lack of ability to measure improvement. |
| Unpredictable clinical management | Intervention or actions triggered by it may be perceived as random by the patient and thus reduces their sense of control. |
| No effect on self-care | Patients feel that the intervention, or actions triggered by the intervention made no difference to their outcome or quality of life, and therefore were not motivated to change self-care behaviour. |
| Lack of improvement | As a result of intervention, no or little improvement in clinical care or symptoms was perceived, due to lack of the intervention's effect. |
| Technical difficulties | Intervention malfunctions while in use, and does not reliably work as intended. |
| Medical jargon | Understanding of the intervention, information, or training phases was disrupted by the presence of medical jargon, which reduces the patient understanding. |
| Not user friendly | Intervention is not intuitive or easily usable by the patient, or is too complex to apply. |
| Lack of portability | The device is not portable and causes issues with patients travelling or on holiday. |
| Technology overwhelming | Patients are not computer literate or technically proficient enough to use the intervention. |
| Lack of training | Lack of training or provided instructions in the use of the device or software, leaving users confused with its application and use. |
| Lack of education | Lack of education on the disease process, what to expect, and reason behind using a new intervention, leading to a reduced view of its importance, and misconceptions about their disease state. |
| Irrelevant training | Patients are given training or education on topics which are not relevant to them or are not flexible enough to encompass their situation. |
| Unreliable information | Information gained due to intervention is unreliable or untrustworthy. |
| Intrusive | Comments that the intervention is bulky, or intrusive in the patient's environment. |
| Cost | The intervention is expensive for the patient. |
| Lack of privacy/ security | Concerns with patient confidentiality and privacy when using remote care. |
| Lack of options | Lack of flexibility, information, or treatment options provided by the intervention, which could have been added to make it more effective. |
| Adds extra concern for carers | The intervention may cause anxiety and insecurity for carers. This may be due to added responsibility, complexity, or the stress of an extra task in their care. |
| Lack of support for care | Lack of home care and support for utilising the intervention at home and looking after themselves, causing concern for how they will cope, and overburdening current carers. |
| Extra work = tiring | The intervention adds extra work to the patient's daily life which can be stressful and time consuming. |
| Uncomfortable at home | Some patients feel that their home environment is uncomfortable during times of illness due to inconveniences such as lack of care, stairs and pain. |
| Lack of efficiency/ co-ordination | Intervention administrators are not co-ordinated enough resulting in inefficient or mistimed service which is an inconvenience. |
| Cannot replace hospital care | The intervention cannot replace the environment of a hospital setting; it is not thorough enough, nor provides the same amount of assurances as constant surveillance, and patients would not feel comfortable using the intervention as an alternative. |
| Extra work for clinicians | Patients are concerned that doctors may be overburdened by extra work brought on by the intervention. |
| Threat to independence/ control | Intervention threatens the sense of independence and control patients have over their lives and daily routine, and makes them feel restricted and tied to a machine. |
| Technology not needed | Current care is already optimal and extra technology is not needed to meet the patient's current health needs. |
| Language barriers | The patient finds it difficult to use the intervention due to a language barrier. |
| **CARER POSITIVE EXPERIENCES** | |
| Feel patient is ‘looked after’ | The perception that the intervention allows the patient to be monitored by healthcare staff more easily and provides a safety net for further problems, which can be reassuring. |
| Extra support | The intervention supports the carer's efforts in caring for the patient in terms of physical, mental or psychological help where it is needed. |
| Informative/ educational | The intervention provides information to help the carer in their daily tasks by making them aware of the symptoms and signs of disease for example to help support their patient. |
| User friendly | The intervention is easy to use and apply by the carer |
| **CARER NEGATIVE EXPERIENCES** | |
| Extra responsibility | The intervention adds extra responsibility to the job of caring for the patient by creating an extra task, which may cause stress if not confident with the intervention. |
| Change is stressful | Carers have a daily routine. The intervention disrupts the daily activities of the carer and forces them to make changes in ways they may not be prepared for. This change adds extra stress to the job of caring. |
| Causes concern for patient | The intervention adds extra concern and anxiety for the carer by creating an extra task, escalating their fear, and propagating the perception that the disease is progressing and needs constant attention to prevent worsening. |
| Control taken away | Carers may feel that the intervention takes away some of the control they are used to over the patient's lives and makes them more reliant on healthcare staff. |
| Invasion of privacy | Intervention may be seen as intrusive, e.g. constant monitoring can threaten the privacy shared between patient and carer. |
| Lack of education | A lack of information about disease management may leave the carer unsure how to react to different situations of care. |
| Lack of improvement | Carers may perceive no improvement in symptoms or clinical status from the intervention for the patient |
| Technical difficulties = stressful | Difficulties caused by malfunction of the intervention can be stressful for carers who rely on it. |
| Not user friendly | The carer finds the intervention difficult to apply or use |
| Technology not the solution | Intervention cannot solve the patient's critical needs and cannot replace the services of usual care such as trained carers; or the patient is too ill to make use of the intervention. |
| **HEALTHCARE STAFF POSITIVE EXPERIENCES** | |
| Familiarity/ communication with patient | Better familiarity with patient norms and tendencies allows relative comparisons and better personalised decision making. Intervention allows the staff to get to know the patient better and so makes management decisions easier. Emphasis on communication and continuity of care. |
| Encourages teamwork | The intervention fosters collaboration between healthcare staff and promotes teamwork and communication for better patient care such as by sharing information easily between professionals. |
| Better knowledge of patient status | Technology allows staff to check in on patients more frequently than regular clinic visits, and gives them a better idea of a patient's condition. This may lead to better decisions. Emphasis on clinical decision making and optimal monitoring. |
| Pro-active management | Technology helps staff predict negative health outcomes and take actions to prevent them sooner. |
| Confidence in management | Remote care that increases frequency of monitoring allows medication to be trialled with a shorter review period, and so increases confidence in decisions of management such as medication changes. |
| Identifies priorities in care | The intervention helps physicians organise information and identifies or presents gaps in care, such as vulnerable patients, or reduced performance, in order to improve quality of care. |
| Improvement from usual care | The intervention is an improvement in the quality of care from usual practice as viewed from a staff perspective. |
| Reduces error | The design of the intervention allows extra care in the management of a patient, reducing the risk of errors in care. |
| Important | A perceived importance of the intervention and the sentiment that the intervention is vital to optimal care of the patient's condition, and is therefore a worthwhile investment. |
| Encourages patient self-care | Staff's perception that the use of the intervention encourages or motivates patients to care for themselves better, or educates them in improving and getting used to their own self-care. |
| Increases patient knowledge | The intervention helps patient by increasing knowledge and providing information about management of their disease state and other useful information to make the patient self-reliant. |
| Staff education | The intervention provides up-to-date information and learning resources for staff and physicians to improve their knowledge and care. |
| User friendly | Easy for clinicians to use. |
| Technical support | Non-clinical support is provided in order to aid integration of the system into current practice. |
| Security/ private | The intervention information is secure, accessible only to staff members it is assigned to, and can be private enough to maintain patient confidentiality. |
| Automated saves time | Automated process can take the workload away from the staff or clinician and allow easier and quicker patient management than usual care. |
| No change in workload | Despite the added intervention, the workload of staff is not increased or is even reduced, allowing more time to prioritise to other patients i.e. the intervention is not disruptive to current work patterns. |
| Incentivisation | The cost of the intervention is offset by incentives which add to the appeal of adopting the intervention such as monetary funding for its use. |
| Saves travel time | The intervention does or could potentially save the patient travel time. |
| Local champions | Recognised local champions for the intervention are appointed to aid and support telehealth. |
| Cost savings | The effect of the intervention is seen to be able to save costs in the long run e.g. by reducing the amount of admissions or critical cases that require further treatment. |
| Flexible to practice | The intervention can be shaped around the healthcare staff’s current practice or adapt to suit different needs as required. |
| **HEALTHCARE STAFF NEGATIVE EXPERIENCES** | |
| Perceived dependence on staff | Greater communication and input allowed by remote care makes staff perceive an increased dependence of patients on their support in their daily lives. |
| Increases patient concern/ confusion | The increased overload of health information and input from the intervention may cause extra concern, worry or confusion for patients who cannot clearly understand. |
| Lack of patient-to-staff communication | Lack of communication links and options for communication between patients and physicians. |
| Lack of staff-to-staff communication | New interventions work on a separate system and do not allow communication and referral of patient details to other practitioners and staff, and thus limits inter-professional communication. |
| Not linked to records | A discord between the new intervention and already established health record systems makes it difficult for staff to adjust to a new system and complicates application. |
| Inflexible guidance | Staff perceive the intervention as directive and controlling of their patients e.g. giving instructions without adaptation or feedback. |
| Not user friendly | Difficult, unintuitive, or overly complex for clinicians to use and adapt for clinical work. |
| Technical difficulties | Problems with the use of technology, or malfunction of any software or hardware used by healthcare professionals. |
| Computer literacy | Poor computer literacy or technical skills of the staff is a barrier to using the intervention. |
| Lack of equipment/ support | A lack of equipment such as computer facilities or technical support prevents the intervention from being adopted widely. |
| Lack of training | Lack of training to use the device or intervention, leading to reduced confidence and knowledge of its application. |
| Lack of education | Lack of patient's education on disease process, or training for use of the intervention. |
| No change in patient self-care | Staff perception that the intervention may lead to lack of motivation in self-care for patients. |
| No improvement | No perceived improvement in the patient's symptoms or clinical health status including mortality or admission rates as a result of the intervention (from the healthcare staff’s perspective). |
| Increased workload | Staff felt that the intervention added to their burden of work by increasing the amount of time required to spend with each patient in the application of the technology, without an increase in compensation. |
| Slow to change practice | Any statements relating to resistance or reluctance to change current established practice. |
| Increases errors | The intervention adds an extra layer to the management of a patient's illness, increasing the likelihood of an error in care. |
| Lack of options | Lack of information, flexibility, treatments and functionality options which could have been added to the intervention to make it more effective. |
| Patient selection decisions | Intervention is not suitable for all patients and requires a decision for selection on behalf of staff. Not all patients would benefit from being selected. |
| Cost | Cost and expense considerations from integrating technology from a healthcare staff perspective. |
| Health inequality | The disparity between the intervention group and the rest of the population who are not able to receive the intervention may create a health inequality gap. |
| Medicolegal concerns | Statements which involve moral or legal concerns, such as legal issues whether clinicians are obligated to respond to warning alerts immediately. |
| Information privacy/ security | Concerns and comments relating to the storage and transfer of confidential patient information. |
| Limits clinical care | The quality of clinical care is not as good with the intervention as with usual care or face-to-face care; More information is felt to be gathered when talking to a patient face-to-face. |
| Lack of confidence in equipment/ readings | Intervention gives information that may be seen as unreliable, reducing clinician trust in the intervention. |
| Clinician anxiety | Intervention increases clinician anxiety due to handing over some of their responsibilities to others or to the device. |
| Lack of evidence of effectiveness | Staff views that there is insufficient evidence to show whether the intervention is effective. |
