# Supplementary material for: Factors Affecting Patient and Physician Engagement in Remote Health Care for Heart Failure: Systematic Review
Source: JMIR Cardio. 2022 Apr 6;6(1):e33366. doi: 10.2196/33366 (PMC9021943; doi:10.2196/33366)
Supplement: Multimedia Appendix 6 [file cardio_v6i1e33366_app6.doc]

**Multimedia Appendix 6**: Synthesis of Second order codes

| |  | **Second order codes** | **First order codes** | | --- | --- | --- | | Positive patient experiences | Improves clinical care | Improvement from normal care | | Better follow up and co-ordination of care | | Patient feels ‘looked after’ | | Increased confidence in staff’s management | | Improves self-care | Increased knowledge of their condition | | Increased confidence and motivation for self-care | | Increased involvement in self-care | | Improved communication | Psychosocial support | | Patient to patient communication | | Accessible Design | User friendly | | Popular with patients | | New technology is novel and exciting | | Adds Convenience | Saves travel time | | Increased comfort and freedom at home | | Intervention is flexible to patient’s lifestyle | | Negative patient experiences | Poor design / Inaccessible to patients | Medical Jargon | | New technology overwhelming | | Intrusive | | Not user friendly | | High Cost | | Technical difficulties | | Lack of portability | | Lack of feedback | | Gives unreliable information | | Lack of privacy and security | | Loss of control | Unpredictable changes in management | | Pt feels uncomfortable at home | | Lack of flexibility | | Intervention threatens independence and control | | Reliance on staff | | Increased burden of work and responsibility | Extra work is tiring | | Lack of extra support to help cope with intervention | | More responsibility causes anxiety | | Extra concern for carers | | Worries about extra work for staff | | Does not contribute to care | No effect on self-care | | Technology not needed | | Cannot replace hospital care | | Lack of improvement from normal care | | Lack of co-ordination and efficiency | | Poor communication | Lack of patient to patient communication | | Lack of patient to staff communication | | Language barriers | | Inadequate patient education | Lack of education on disease and health condition | | Lack of training for intervention | | Irrelevant training for intervention | |
| --- | --- | --- | --- | --- | --- | --- | --- | --- | --- | --- | --- | --- | --- | --- | --- | --- | --- | --- | --- | --- | --- | --- | --- | --- | --- | --- | --- | --- | --- | --- | --- | --- | --- | --- | --- | --- | --- | --- | --- | --- | --- | --- | --- | --- | --- | --- | --- | --- | --- | --- | --- | --- | --- | --- | --- | --- | --- | --- | --- | --- | --- | --- |

| |  | **Second order codes** | **First order codes** | | --- | --- | --- | | Positive carer experiences | Improved care | Feels the patient is ‘looked after’ | | Leads to extra support | | Improves Education | Educational and informative | | Accessible Design | User friendly | | Negative carer experiences | Increases stress of care | Change in routine is stressful | | Extra responsibility | | Adds extra concerns for patient and carer | | Technology is not needed | Technology is not enough | | Lack of improvement | | Poor education | Lack of education | | Inaccessible design | Not user friendly | | Technical difficulties are stressful | | Loss of control | Invasion of privacy | | Control is taken away | |
| --- | --- | --- | --- | --- | --- | --- | --- | --- | --- | --- | --- | --- | --- | --- | --- | --- | --- | --- | --- | --- | --- | --- | --- | --- | --- | --- | --- |

| |  | **Second order codes** | **First order codes** | | --- | --- | --- | | Positive healthcare professional experiences | Improved communication | Encourages teamwork within staff | | Familiarity and communication with patient | | Accessible Design | Maintains privacy and security | | User friendly | | Incentives provided | | Cost savings | | Technical support provided | | Flexible to practice | | Saves Time | Automated process saves time | | No change in workload | | Saves travel time for patients | | Enhances Quality of Care | Reduces error | | Identifies priorities in patient care | | Improves on normal care | | Promotes pro-active disease management | | Better knowledge of patient’s disease status | | Increases confidence in clinical decisions | | Provides education for staff | | Intervention feels important to staff | | Empowers patients | Creates local champions to promote intervention | | Encourages patient self-care | | Increases patient knowledge | | Negative healthcare professional experiences | Poor communication | Lack of patient to staff communication | | Lack of staff to staff communication | | Does not improve clinical care | Reduces clinical care | | Increases staff errors | | No improvement in patient health status | | Lack of evidence for effectiveness | | No change in patient self-care | | Disruptive to current practice | Increased workload | | Resistance to change | | Handing over responsibility of care causes anxiety | | Requires patients to be ‘selected’ | | Not linked with existing health records | | Poor Design / Inaccessible to staff | High cost | | Medico-legal concerns | | Patient information security or privacy concerns | | Technical difficulties | | Not user friendly | | Poor staff computer literacy | | Lack of equipment or technical support | | Lack of training for intervention | | Lack of confidence in equipment and readings | | Reduces patient independence | Perceived patient dependence on staff | | Lack of options/flexibility | | Intervention gives inflexible guidance | | Poor Patient Education | Lack of education for the patient | | Increases patient confusion and concerns about condition | |
| --- | --- | --- | --- | --- | --- | --- | --- | --- | --- | --- | --- | --- | --- | --- | --- | --- | --- | --- | --- | --- | --- | --- | --- | --- | --- | --- | --- | --- | --- | --- | --- | --- | --- | --- | --- | --- | --- | --- | --- | --- | --- | --- | --- | --- | --- | --- | --- | --- | --- | --- | --- | --- | --- | --- | --- | --- | --- | --- | --- | --- | --- | --- | --- | --- |
